# Specific Cooperation Between Imp-α2 and Imp-β/Ketel in Spindle Assembly During *Drosophila* Early Nuclear Divisions

**DOI:** 10.1534/g3.111.001073

**Published:** 2012-01-01

**Authors:** Erika Virágh, Mátyás Gorjánácz, István Török, Tolga Eichhorn, Sowjanya Kallakuri, Tamás Szlanka, István Kiss, Bernard M. Mechler

**Affiliations:** * Department of Developmental Genetics and; ‡ Department of Pharmaceutical Biology, DKFZ-ZMBH Allianz, Heidelberg 69120, Germany; † Institute of Genetics, Biological Research Center, Hungarian Academy of Sciences, Szeged H-6726, Hungary

**Keywords:** *Drosophila*, Importins, genetic interaction, mitosis, spindle formation

## Abstract

The multifunctional factors Imp-α and Imp-β are involved in nuclear protein import, mitotic spindle dynamics, and nuclear membrane formation. Furthermore, each of the three members of the Imp-α family exerts distinct tasks during development. In *Drosophila melanogaster*, the *imp-α2* gene is critical during oogenesis for ring canal assembly; specific mutations, which allow oogenesis to proceed normally, were found to block early embryonic mitosis. Here, we show that *imp-α2* and *imp-β* genetically interact during early embryonic development, and we characterize the pattern of defects affecting mitosis in embryos laid by heterozygous *imp-α2^D14^* and *imp-β^KetRE34^* females. Embryonic development is arrested in these embryos but is unaffected in combinations between *imp-β^KetRE34^* and null mutations in *imp-α1* or *imp-α3*. Furthermore, the *imp-α2^D14^*/*imp-β^KetRE34^* interaction could only be rescued by an *imp-α2* transgene, albeit not *imp-α1* or *imp-α3*, showing the exclusive *imp-α2* function with *imp-β*. Use of transgenes carrying modifications in the major Imp-α2 domains showed the critical requirement of the nuclear localization signal binding (NLSB) site in this process. In the mutant embryos, we found metaphase-arrested mitoses made of enlarged spindles, suggesting an unrestrained activity of factors promoting spindle assembly. In accordance with this, we found that Imp-β^KetRE34^ and Imp-β^KetD^ bind a high level of RanGTP/GDP, and a deletion decreasing RanGTP level suppresses the *imp-β^KetRE34^* phenotype. These data suggest that a fine balance among Imp-α2, Imp-β, RanGTP, and the NLS cargos is critical for mitotic progression during early embryonic development.

The Ran pathway plays a central role in interphase cells by mediating and regulating the nucleocytoplasmic protein transport (Görlich *et al.* 1996; Izaurralde *et al.* 1997; Stewart 2007). During mitosis, it regulates spindle assembly, metaphase chromosome alignment, and nuclear envelope (NE) assembly (Carazo-Salas *et al.* 2001; Caudron *et al.* 2005; Zhang and Clarke 2000). In all these processes, the same basic mechanism is operational (Dasso 2001): Importin-β (Imp-β) binds to Importin-α (Imp-α) and induces a conformational change opening the NLS-binding site of Imp-α (Harreman *et al.* 2003; Kobe 1999). The NLS-bearing proteins, as cargos for the nuclear import (Mans *et al.* 2004; Pemberton and Paschal 2005) or spindle assembly factors (SAF), and other proteins regulating the dynamics of mitosis (Gruss *et al.* 2001; Nachury *et al.* 2001; Wiese *et al.* 2001) are bound to the Imp-α/Imp-β heterodimer. RanGTP present at a high concentration in the nucleus and distributed along a concentration gradient around the mitotic chromosomes binds to Imp-β and dissociates the complex, thereby liberating the bound proteins (Görlich *et al.* 1996; Walczak and Heald 2008). The RCC1/RanGEF, which mediates the exchange of the Ran-bound GDP for GTP, is associated with the chromatin and hence responsible for the high RanGTP concentration in the interphase nucleus or around the mitotic chromosomes (Nemergut *et al.* 2001). Therefore, the liberation of NLS-bearing proteins occurs in the nucleus or near the chromatin (Bastiaens *et al.* 2006).

The regulation of the SAF activity by the Ran system during mitosis occurs in all eukaryotic organisms, from plants (Jeong *et al.* 2005; Pay *et al.* 2002) and yeast (Fleig *et al.* 2000; Sato and Toda 2007) to humans (Li and Zheng 2004; Moore *et al.* 2002), and it also takes place in eggs like those of *Drosophila* and *Xenopus*, in which large amounts of SAFs and other mitotic proteins are deposited. These factors, including TPX2 (Gruss *et al.* 2001; Schatz *et al.* 2003; Vos *et al.* 2008), NuMa (Merdes *et al.* 1996; Nachury *et al.* 2001; Wiese *et al.* 2001), and NuSAP in the frog (Raemaekers *et al.* 2003; Ribbeck *et al.* 2007), as well as Mars in the fruit fly (Tan *et al.* 2008), are kept inactive under strict spatial and temporal control as abnormal activation are fatal to the embryo. Furthermore, the respective binding affinities of the various SAFs toward the NLS-binding domain of Imp-α appear to be critical to the mitotic process (Hodel *et al.* 2006; Riddick and Macara 2005). Interestingly, in *Drosophila*, where the first 13 rounds of synchronous mitoses take place in a syncytium, the Anillin and Peanut proteins, which are needed to keep the spindles separated, also appears to be regulated by Imp-α/Imp-β and Ran (Silverman-Gavrila and Wilde 2006). Furthermore, the Ran system is required for the assembly and integrity of the NE in eukaryotic organisms (Askjaer *et al.* 2002; Ryan *et al.* 2003; Timinszky *et al.* 2002; Zhang and Clarke 2000). In addition, the Ran pathway exerts a critical role in centrosome duplication (Di Fiore *et al.* 2004), as Ran localizes to centrosomes, partly in the GTP-bound form (Keryer *et al.* 2003).

Phylogenetic studies of higher eukaryotes indicated that the *imp-α* genes could be classified in three conserved clades designated as *α1*, *α2*, and *α3*, whereas the *imp-β* gene is unique (Goldfarb *et al.* 2004; Hogarth *et al.* 2006; Köhler *et al.* 1997; Köhler *et al.* 1999; Malik *et al.* 1997). The first member of the *imp-α* gene family identified in *Drosophila* is *imp-α2* (Török *et al.* 1995), and genetic analysis shows that a loss-of-function mutation in this gene leads to female sterility characterized by the occlusion of the ring canals linking the nurse cells to the oocyte. This occlusion prevents the transfer of cellular components at the time of nurse cell dumping into the oocyte and results in the formation of short basket-type eggs (Gorjánácz *et al.* 2002). In the male gonads, each of the three *Drosophila* *imp-α* genes displays overlapping patterns of expression, and their mutations affect sperm formation (Giarrè *et al.* 2002). Interestingly, overexpression of each of the *imp-α* homologs could restore normal spermatogenesis in any mutated *imp-α* gene, whereas only the expression of an *imp-α2* transgene could restore fertility in mutated *imp-α2* females (Giarrè *et al.* 2002; Gorjánácz *et al.* 2002; Gorjánácz *et al.* 2006; Mason *et al.* 2002; Máthé *et al.* 2000; Ratan *et al.* 2008). However, the role of each *imp-α* genes during embryogenesis is not yet understood. Microinjection of large amounts of Imp-α and Imp-β into *Drosophila* embryos affects spindle assembly and chromosome segregation (Silverman-Gavrila and Wilde 2006). In contrast, functions have been attributed to the *imp-β* gene on the basis that its dominant female sterile mutation *Ket^D^* (Erdélyi *et al.* 1997; Lippai *et al.* 2000) blocks the first gonomeric division and NE assembly (Tirián *et al.* 2000; Timinszky *et al.* 2002).

By making use of various *imp-α* mutants and *Ket^RE34^* or *imp-β^KetRE34^*, a suppressor mutant of *Ket^D^* allowing development to proceed, we studied the roles played by these genes during the syncytial divisions in the *Drosophila* embryo (for the sake of clarity, we denote *Ket^D^* and its recessive revertant alleles as *imp-β* with designation of the *Ket* alleles in superscript). Our genetic approach revealed that *imp-α2* and *imp-β* cooperate to implement spindle dynamics during *Drosophila* early embryonic mitosis. This regulation involves a well-balanced cooperation between the Imp-α2 and Imp-β proteins. Our results clearly demonstrate that *imp-α2* exerts a major role in spindle dynamics that cannot be substituted by *imp-α1* or *imp-α3*. Our biochemical analysis showed that a reduced activity of the Imp-α2/Imp-β complex, resulting from amino acid substitutions in the NLS-binding domain of Imp-α2 or specific modifications in Imp-β, led to a nonspecific release of NLS cargos, causing mitotic defects in preblastoderm embryos.

## Materials and Methods

### Fly stocks

Flies were maintained at 25° on standard cornmeal-yeast-agar medium. Crosses were performed using standard genetic techniques. The following *imp-α2* stocks and transgenic lines were used: *imp-α2^D14^* (Török *et al.* 1995), *P{imp-α2^+^}*, *P{UAS-imp-α2^cDNA^}* (Gorjánácz *et al.* 2002), *P{UAS-imp-α2^ΔIBB^}*, *P{UAS-imp-α2^NLSB^*^−^*}*, *P{UAS-imp-α2^SNLSB^*^−^*}*, and *P{UAS-imp-α2^CASB^*^−^*}* (Gorjánácz *et al.* 2006). The mutant lines *imp-β^KetRE34^*, *imp-β^KetRX13^*, and *imp-β^KetRP13^* (Erdélyi *et al.* 1997) and *P{imp-β^+^}* (Lippai *et al.* 2000) were kindly provided by J. Szabad. The third chromosomal ovarian driver line *P{GAL4VP16-nos.UTR}CG6325^MVD1^*, or *nos-Gal4^VP16^*, the *P*-element insertion lines *w^1118^; RanGap^EP1173^/CyO* and *y w; P{EPgy2}RanGap^EY21763^* and the deficiency *Df(3L)w5.4/TM6*, *Tb* were obtained from the Bloomington Drosophila Stock Center (Indiana University). The stocks *w^1118^*; *imp-β^c02473^*/*CyO*, *w^1118^*; *imp-β^e02657^*/*CyO* and *w^1118^*; *imp-β^e03750^*/*CyO* (Thibault *et al.* 2004) were provided by Exelixis, Harvard Medical School. The RNAi gene silencing constructs *P{imp-α1i^28921^}*, *P{imp-α2i^34265^}*, *P{imp-α2i^34266^}*, *P{imp-α3i^36103^}*, and *P{imp-α3i^36104^}* (Dietzl *et al.* 2007) were obtained from the Vienna Drosophila RNAi Center. The lines *w^1118^; Df(3L)α1S1 ca/TM6B* uncovering the *imp-α1* gene (Ratan *et al.* 2008) and *imp-α3^17-7^/TM3(KR-GFP*), *Sb*, as well as flies carrying the *UTR^Δ^*-*imp-α* constructs *P{UTR^Δ^*-*imp-a1}*, *P{UTR^Δ^-imp-a2}*, and *P{UTR^Δ^-imp-a3}* inserted on the second chromosome (Mason *et al.* 2003), were kindly provided by R. J. Fleming. A *nos-Gal4^VP16^*, *P{UAS-imp-α2^cDNA^}* chromosome was generated by meiotic recombination. Recombinants were selected according to their stronger eye color and verified by PCR using primer pairs specific for each transgene. The *pUASp2*-based plasmid carrying the zz-tagged *imp-α2* sequences (see below), were microinjected along with the *Δ23* transposase helper plasmid into *w^1118^* syncytial blastoderm embryos according to standard techniques, and stable lines were generated.

### Embryo viability

Females with different allele combinations were collected as virgins, and 20–30 of them were mated to 30 Oregon-R males. Eggs were collected from 3–6-day-old mothers for 12 hr on fresh apple juice plates (22.5 g agar boiled in 750 ml distilled water, mixed with 25 g sucrose and 250 ml apple juice) supplemented with charcoal, at 25°. The plates were incubated at 25° for an additional 28 hr, and the percentage of empty eggshells was determined with respect to the total numbers of laid eggs. All experiments were repeated three times.

### DNA sequencing of *imp-β^KetRE34^*

Genomic DNA was isolated from a single homozygous *imp-β^KetRE34^* second instar larva according to Gloor *et al.* (1993), and 1 μl of the preparation was used for PCR reaction in a 25 μl volume. A region (376–4122 bp, according to FlyBase numbering) of *imp-β* gene, covering the whole coding sequence, was PCR-amplified in two overlapping reactions using High-Fidelity PCR Master Kit (Roche Applied Science). For the first segment, we used the forward primer 376 (5′-TCCATCACCCACACAGACGCAC-3′) starting 83 bp before the ATG translation initiation site and the reverse primer 2632 (5′-TATGTCTCGTTGATAGCCGCCTCG-3′), whereas for the second segment, ending 134 bp after the termination codon, we used the forward primer 2424 (5′-CTTAAAGCCGCTCGTGGAGCAAG-3′) and the reverse primer 4122 (5′-CAAGAATCGACACACCATTCGTTC-3′). The amplified products were separated on an agarose gel and isolated with the help of a QIAGEN DNA Purification Kit according to the manufacturer’s instruction, and sequenced on an ABI 3730XL DNA sequencer.

### Embryo fixation and immunohistochemistry

Eggs were collected on apple juice plates for 2 hr at 25° and incubated for additional 2 hr. After dechorionization in 4% bleach for 4 min, the eggs were rinsed consecutively in H_2_O, 0.2% Triton X-100, and H_2_O. For methanol fixation, the embryos were shaken vigorously for 45 sec in a 1:1 mixture heptane:methanol at room temperature and rinsed three times in methanol for 5 min each. Fast formaldehyde fixation and immunostaining of either methanol- or formaldehyde-fixed embryos were performed according to standard procedures (Rothwell and Sullivan 2000). Primary antibodies used were rat anti–α-tubulin YL1/2 (1:400, Serotec), rabbit anti-centrosomin [1:200 (Heuer *et al.* 1995), kindly provided by T. C. Kaufman], mouse anti-lamin Dm0 [1:30, (Paddy *et al.* 1996), a gift of H. Saumweber], and rabbit anti-phosphohistone (1:500, Santa Cruz Biotechnology). Secondary antibodies Alexa Fluor 488 anti-mouse (1:300) and Alexa Fluor 488 anti-rabbit (1:500) were purchased from Invitrogene and Cy3 anti-rat (1:400) from Jackson ImmunoResearch Laboratories. DNA was stained with DAPI. The samples were examined with a Nikon C1Si-CLEM confocal laser scanning microscope of the Nikon Imaging Center at the University of Heidelberg.

### Protein A tagging of Imp-α2 proteins

In the first step, wild-type and NLSB^−^ mutant *imp-α2* cDNAs (Gorjánácz *et al.* 2006) were PCR-amplified with the forward primer 5′-ATAAGAATGCGGCCGCCACACATTTCATCGCAGCAGCAAAC-3′ and the reverse primer 5′-CCCAAGCTTGAACGTGTAGCCACCCTCGGGAGCC-3′ including extensions of *Not*I and *Hin*dIII recognition sites, respectively. After digestion with the appropriate enzymes, PCR products were cloned into *pBluescript II SK(−*) vector digested with *Not*I and *Hin*dIII. In a second step, the IgG-binding domain (zz tag) of Protein A was PCR-amplified from the *pBS1479* TAP-tagging vector (Puig *et al.* 2001; Rigaut *et al.* 1999) with the forward primer 5′-CCCAAGCTTAAAACCGCGGCTCTTGCGCAACACG-3′ and the reverse primer 5′-CGGGGTACCTTATCAGGTTGACTTCCCCGCGGAATT-3′ containing extensions of *Hin*dIII and *Kpn*I recognition sites, respectively. The PCR product was digested with *Hin*dIII and *Kpn*I, and then cloned into the *Hin*dIII and *Kpn*I sites of the *imp-α2* plasmids generated in the first step. Finally, the zz-tagged *imp-α2* sequences were cut out with *Not*I and *Kpn*I and then cloned into the *pUASp2*-transforming vector digested with the same enzymes. All PCR products were sequenced to confirm the absence of PCR-induced errors. Molecular cloning techniques were performed according to standard procedures (Sambrook and Russell 2001). Enzymes were obtained from Promega Biotech.

### Purification of Imp-α2 protein complexes

Females expressing in their ovaries the zz-tagged forms of wild-type or NLSB^−^ Imp-α2 proteins were dissected on ice, and ovaries were collected in ice-cold binding buffer (50 mM Tris, pH 7.5, 100 mM NaCl, 20 mM KCl, 3 mM MgCl_2_, 5% glycerol, 0.1% NP40, 0.5 mM PMSF, and protease inhibitors). All subsequent manipulations were done at 4°. To obtain a high-speed supernatant (HSS), ovaries were homogenized and centrifuged first with 10,000 *g* and then with 100,000 *g* for 60 min. IgG Sepharose beads (6 Fast Flow, GE Healthcare Life Sciences) were added to the HSS and incubated for 16 hr. The beads were then sedimented and washed several times with binding buffer. The proteins bound to IgG Sepharose beads were eluted with 50 mM Tris, pH 7.5, and 2 mM MgCl_2_. The purified proteins were separated on SDS-PAGE, stained with Coomassie Brilliant Blue, and then proteins in selected bands were identified by MALDI spectroscopy in the Department of Proteome Analysis by M. Schnölzer at the German Cancer Research, Heidelberg.

### *In vitro* mutagenesis and expression of mutant proteins in bacteria

Wild-type *imp-β* cDNA (Lippai *et al.* 2000), kindly provided by J. Szabad, was cloned in *pBluescript II SK(+*) vector, and mutant constructs were generated using the PCR-based QuickChange Site-Directed Mutagenesis Kit (Stratagene) according to the manufacturer’s instructions. In the first step, the P^446^L mutation of the dominant female sterile *imp-β^KetD^* allele was generated with PCR primers (bold letters indicate the introduced nucleotide substitutions) 5′-CGGACGTATTTGCGATATAATTC**T**CGAGGCGGCTATCAACG-3′ and 5′-CGTTGATAGCCGCCTCG**A**GAATTATATCGCAAATACGTCCG-3′. In the second step, the D^725^N mutation of *imp-β^KetRE3^*^4^ allele was introduced using the P^446^L construct as template with the primers 5′-GGTTCTGTCTGCTTTCGGA**A**ATATTGCGTTGAGC-3′ and 5′-GCTCAACGCAATAT**T**TCCGAAAGCAGACAGAACC-3′. Both constructs were sequenced to confirm the absence of PCR-induced errors. Sequences containing the full-length open reading frame of the *imp-β^KetD^* and *imp-β^RE3^*^4^ alleles and the wild-type *imp-β* coding sequence were cloned in frame into the *pGEX-4T-2* expression vector (GE Healthcare Life Sciences) to create GST-fusion constructs. Fusion proteins were expressed in BL21-CodonPlus cells (Stratagene) at room temperature and purified on Glutathione Sepharose beads (GE Healthcare Life Sciences) according to the manufacturer’s instructions.

### GST-pulldown experiments

Bacterially expressed and purified His-RanT^24^N (RanGDP form of Ran) or His-RanQ^69^L (RanGTP form of Ran) proteins of *Xenopus* (kindly provided by I. W. Mattaj) were diluted to a final concentration of 10 μM in a protein extract of 2-hr-old embryos. Embryonic extracts were prepared in IP buffer (10 mM TrisHCl, pH 7.5, 50 mM KCl, 0.1% Tween 20, protease inhibitors) at a concentration of 0.2 g embryo/ml as described before (Máthé *et al.* 2000). In a 0.5 ml tube, 0.4 ml aliquots of the above mixtures were incubated with 80 μl of a suspension made of GST-Imp-β, GST-Imp-β^KetD^, or GST-Imp-β^KetRE34^ fusion proteins bound to Glutathione Sepharose beads for 60 min at room temperature. The beads were then washed extensively in IP buffer and directly suspended in 2X SDS loading buffer. Proteins bound to the beads were separated by SDS-PAGE and detected in Western blot experiments with rabbit anti–Imp-β antibodies (1:2000) provided by J. Szabad (Lippai *et al.* 2000) and rabbit anti-Ran (H-96) (1:200, Santa Cruz Biotechnology) antibodies.

## Results

### Genetic analysis of the *imp-α2* and *imp-β* interaction

To determine whether any combination between mutations in the *imp-α2* and *imp-β* genes could result in a synthetic phenotype, we first combined six different recessive *imp-β* alleles (Table 1) with the interstitial deficiency *imp-α2^D14^* (Gorjánácz *et al.* 2002; Török *et al.* 1995) and examined the viability of the eggs laid by heterozygous females of each combination. In all experiments, mutant females were crossed to wild-type males, and the hatched larvae were scored as a percentage of the total laid eggs. As shown in Table 1, eggs produced by *trans*-heterozygous *imp-a2^D14^*/*imp-β^KetRE34^* females are lethal, whereas eggs laid by all other heterozygous females develop normally.

**Table 1:** Viability of eggs laid by mutant females

Female Genotype	Egg Viability (%)	SD	*n*
*imp-α2^D14^/+*	89	2.49	285
*imp-β^KetRE34^/+*	61	9.50	234
*imp-α2^D14^/imp-β^KetRE34^*	0	0.00	∼10,000
*imp-β^KetRX13^/+*	79	3.23	120
*imp-α2^D14^/imp-β^KetRX13^*	80	9.97	300
*imp-β^KetRP13^/+*	85	5.24	150
*imp-α2^D14^/imp-β^KetRP13^*	85	9.29	276
*imp-β^c02473^/+*	93	2.82	100
*imp-α2^D14^/imp-β^c02473^*	90	5.56	290
*imp-β^e02657^/+*	83	1.41	100
*imp-α2^D14^/imp-β^e02657^*	90	2.88	150
*imp-β ^e03750^/+*	92	6.24	275
*imp-α2^D14^/imp-β^e03750^*	94	3.44	175

For the origin of mutant *imp-β* alleles, see *Materials and Methods*.

*n*, number of embryos scored.

Genomic *P{imp-α2^+^}* or *P{imp-β^+^}* transgenes or a *P{UAS-imp-α2^cDNA^}* transgene driven by *nos-Gal4^VP16^* could significantly restore embryonic development of eggs laid by heterozygous *imp-a2^D14^/imp-β^KetRE34^* females (Table 2), indicating that the observed interaction involved no second site mutation carried on either the *imp-a2^D14^* or the *imp-β^KetRE34^* chromosome. Further, we tested whether RNAi silencing of *imp-α2* would also produce a similar phenotype with *imp-β^KetRE34^*. Expression of the *P{imp-α2i^34266^}* construct (Dietzl *et al.* 2007) driven by *nos-Gal4^VP16^* apparently exerted no effect on a wild-type background, but it reduced embryonic viability in eggs laid by heterozygous *imp-α2^D14^* females (supporting information, Table S1). We obtained an even more dramatic effect when *P{imp-α2i^32466^}* was expressed in females homozygous for wild-type *imp-α2* but heterozygous for *imp-β^KetRE34^*. Only ∼3% of the eggs were viable. These data show that a significant reduction of *imp-α2* expression in the ovary in combination with *imp-β^KetRE34^* resulted in high percentage of lethality of embryos laid by these females, indicating that the coordinated action of the Imp-α2 and Imp-β proteins is critical during embryogenesis.

**Table 2:** Increased *imp-α2 or imp-β* gene dosage restores viability of eggs laid by *imp-α2^D14^/imp-β^KetRE34^* females

Female Genotype	Egg Viability (%)	SD	*n*
*imp-α2^D14^/imp-β^KetRE34^*	0	0.00	∼10,000
*imp-α2^D14^/imp-β^KetRE34^; P{UAS-imp-α2^cDNA^}/nos-Gal4^VP16^*	38	7.77	280
*imp-α2^D14^/imp-β^KetRE34^; P{imp-α2^+^}/+*	46	2.30	340
*imp-α2^D14^/imp-β^KetRE34^; P{imp-β^+^}/+*	30	2.12	172

*n*, number of embryos scored.

The *imp-β^KetRE34^* allele is an EMS-induced recessive revertant of the P^446^L substitution-characterized, dominant negative female-sterile *imp-β^KetD^* (Erdélyi *et al.* 1997; Lippai *et al.* 2000; Timinszky *et al.* 2002; Tirián *et al.* 2000). As no apparent defect could be detected in the-combination between *imp-α2^D14^* and either *imp-β^KetRP13^* (*P*-element-induced recessive revertant) or *imp-β^KetRX13^*, which is an X-ray–induced null allele of *imp-β^KetD^* (Lippai *et al.* 2000), we presumed that *imp-β^KetRE34^* should carry an additional intragenic mutation.

Therefore, we determined the nucleotide sequence of the coding region in *imp-β^KetRE34^*. Besides the nucleotide modification leading to the substitution P^446^L, we found an additional nucleotide change resulting in the substitution of an aspartic acid at position 725 by an asparagine. Therefore, the D^725^N substitution could be a good candidate for the mutation that partially abrogates the dominance of *imp-β^KetD^*, although we could not exclude that an additional mutation may have taken place in the promoter or UTR region. Such a mutation could affect the synthesis of the Imp-β^KetRE34^ protein and weaken the dominant negative phenotype.

### *In silico* analysis of the molecular structure of Imp-β^D725N^

As the D^725^N substitution is located in the Imp-α–binding domain of Imp-β, we performed an *in silico* analysis through docking of the IBB domain of Imp-α2 on Imp-β^D725N^ to determine whether the substitution would markedly change the interaction between both Imp proteins. We found no alteration in the binding affinity between these molecules (Table S2). Further analysis of the modeled structure revealed that residue N^725^ located in the Helix B of HEAT repeat 16 forms an intramolecular polar interaction with residue E^773^ in Helix B of HEAT repeat 17 (Figure S1). This interaction allows less sliding of the B helices in the repeats 16 and 17 along each other, stabilizing in this way their relative positions. Imp-β is known to go through extensive conformational changes during its binding cycle (Conti *et al.* 2006), displaying the closest conformation when bound with the IBB domain of Imp-α. For homology modeling, we used the Protein Data Bank (PDB) data of the human Imp-β captured in association with the IBB domain of human Imp-α [PDB code 1QGK, (Cingolani *et al.* 1999)]. It is possible that the D^725^N substitution might fix a closed structure by making the conformation of the Imp-β^KetRE34^ less prone to be open. This could compensate, at least to some extent, for the opening effect of the P^446^L substitution of the dominant negative Imp-β^KetD^ reported earlier (Timinszky *et al.* 2002) and may thus reduce Imp-β^KetRE34^ toxicity.

### *imp-α2* critical function during early embryonic development

To determine whether the other two members of the *imp-α* gene family would interact with *imp-β*, we combined classical alleles and RNAi knockdowns of *imp-α1* or *imp-α3* with a heterozygous *imp-β^KetRE34^*. As shown in Table 3, the deficiency *Df(3L)α1S1*, uncovering *imp-α1* (Ratan *et al.* 2008), moderately reduced egg viability (47%, compared with 61% for eggs laid by *imp-β^KetRE34^/+* females). The *imp-α3^17-7^* mutant allele producing a polypeptide of 131 residues (Mason *et al.* 2003) decreased the egg viability to 33%. RNAi silencing of either *imp-α1* or *imp-α3* resulted in very similar hatching ratios (40% and 43%, Table S3). These observations indicate that a decrease of the maternal “dowry” of either Imp-α1 or Imp-α3 produced only a moderate reduction of embryonic viability in the *imp-β^KetRE34^/+* background. In contrast, in combination with *imp-β^KetRE34^*, a decrease in Imp-α2 caused either by a deletion or by RNAi silencing resulted in strong embryonic lethality.

**Table 3:** Effects of reduced gene dosages of the three *imp-α* genes on embryo viability in combination with *imp-β^KetRE34^*

Female Genotype	Egg Viability (%)	SD	*n*
*imp-β^KetRE34^/+*	61	9.50	234
*imp-α2^D14^/imp-β^KetRE34^*	0	0.00	∼10 000
*imp-β^KetRE34^/+; Df(3L)α1S1/+*	47	2.36	230
*imp-β^KetRE34^/+; imp-α3^17-7^/+*	33	7.07	181

Deletion *Df(3L)α1S1* uncovers the *imp-α1* gene.

*n*, number of embryos scored.

As the amount of Imp-α2 is apparently higher in eggs than the amount of Imp-α1 or Imp-α3, the overall NLS-binding capacity is mainly ensured by Imp-α2. Therefore, a decrease in the expression of *imp-α2* results in a stronger reduction of this capacity than does a decrease in the expression of *imp-α1* or *imp-α3*, and it could also have a stronger effect on embryonic development. This prompted us to examine whether the effect on hatching rates was independent of the relative amount of the different Imp-α proteins deposited in the eggs. For this purpose, we tested transgenes producing similar amounts of Imp-α, in which the 5′ and 3′ UTR sequences of the various *imp-α* cDNAs were removed and contained an initiation AAAATG consensus sequence (Cavener 1987) inserted at the 5′ extremity of the coding region (Mason *et al.* 2003). As shown in Table 4, only *P{UTR^Δ^*-*imp-α2}* was able to restore embryonic viability in an *imp-α2^D14^/imp-β^KetRE34^* background. The rescue was, however, lower (21%) than with a full *P{imp-α2^cDNA^}* construct (38%, Table 2), indicating the relative importance of UTR sequences in the expression of the *imp-α2* gene. Accordingly, in control experiments, we found that the *P{UTR^Δ^*-*imp-α2}* expressed in the germ line of *imp-α2^D14^* females resulted in 37% embryonic viability, whereas a full-length cDNA construct yielded 85% viability (detailed data not shown). These data indicate that, during early embryonic development when the maternally deposited proteins are the limiting factors, Imp-α2 acts critically in cooperation with Imp-β and could not be substituted with Imp-α1 or Imp-α3.

**Table 4:** Effects of the three different *P{UTR^Δ^-imp-α}* constructs on the viability of eggs laid by *imp-α2^D14^/imp-β^KetRE34^* females

Female Genotype	Egg Viability (%)	SD	*n*
*imp-α2^D14^/imp-β^KetRE34^*	0	0.00	∼10 000
*P{UTR^Δ^-imp-α1}*, *imp-α2^D14^/imp-β^KetRE34^; nos-Gal4^VP16^/+*	0	0.00	1426
*P{UTR^Δ^-imp-α2}*, *imp-α2^D14^/imp-β^KetRE34^; nos-Gal4^VP16^/+*	21	2.08	355
*P{UTR^Δ^-imp-α3}*, *imp-α2^D14^/imp-β^KetRE34^; nos-Gal4^VP16^/+*	0	0.00	1350

*n*, number of embryos scored.

### Specific substitutions in the NLSB domain of Imp-α2 produce embryonic lethality in combination with a reduced *imp-β* gene dosage

As a strong interaction between *imp-α2* and *imp-β* was detected in eggs laid by *imp-α2^D14^/imp-β^KetRE34^* females, we determined which domain of *imp-α2* should be altered to produce embryonic lethality when the *imp-β* gene dosage is reduced. Heterozygous combinations of *imp-α2^D14^* with distinct *imp-β^0^* alleles, including the interstitial deficiency *imp-β^KetRX13^*, and the *P*-element–induced *imp-β^KetRP13^* mutation (Erdélyi *et al.* 1997), as well as the *piggyBac* insertion mutations *imp-β^c02473^*, *imp-β^e02657^*, and *imp-β^e03750^* (Thibault *et al.* 2004), should provide us with a sensitized genetic background for testing four previously modified *imp-α2* constructs (Gorjánácz *et al.* 2006).

In *imp-α2^D14^* homozygous background the *NLSB*^−^ construct (in which the conserved W and N residues of the major and minor NLSB sites are substituted by A) and the *CASB*^−^ construct (in which the six GLDKLE residues of the CAS nuclear export factor binding site are replaced similarly) exert a toxic effect during oogenesis. In contrast, the *SNLSB*^−^ construct, which contains substitutions of A in the conserved W and N residues of the small NLSB site, and the *ΔIBB* construct, in which the Imp-β–binding domain is deleted, restore oogenesis but block embryogenesis under the same conditions (Gorjánácz *et al.* 2006). All four mutated *imp-α2* cDNA constructs contain a *UAS* promoter. These cDNA constructs were expressed in an *imp-α2^D14^/imp-β^0^* background driven by *nos-Gal4^VP16^*. Viability of the eggs laid by these females was measured.

We found that the expression of both *P{UAS-imp-α2^NLSB^*^−^*}* and *P{UAS-imp-α2^SNLSB^*^−^*}* completely blocked embryonic development in eggs laid by all sensitized *trans*-heterozygous females with exception of those with *imp-β^KetRP13^*, indicating that this *P*-element–induced allele is a hypomorph. In contrast, the expression of *P{UAS-imp-α2^ΔIBB^}* and *P{UAS-imp-α2^CASB^*^−^*}* exerted no deleterious effect on embryonic development (Table 5). The ineffectiveness of *P{UAS-imp-α2^ΔIBB^}* can be explained by a lack of the IBB domain, which prevents binding to Imp-β, whereas the other three Imp-α2 proteins contain an intact IBB domain and were able to physically interact with Imp-β. Interestingly the antimorphic effect of *P{UAS-imp-α2^NLSB^*^−^*}* could already be detected in *imp-α2^D14^/+* females, and this effect was enhanced when the *imp-β* gene dosage was reduced. Altogether, our data indicate that the NLSB domain, albeit not the CASB domain, mediates the genetic interaction between *imp-α2* and *imp-β*. We further conclude that the cooperation between Imp-α2 and Imp-β requires the binding of one or several NLS-containing factors involved in the regulation of early embryonic mitosis.

**Table 5:** Expression of mutant *imp-α2* with an inactive NLS-binding domain strongly reduced egg viability laid by heterozygous *imp-α2^D14^/imp-β^0^* females

Female Genotype*^a^*	Egg Viability (%)			
	*NLSB^−^*	*SNLSB^−^*	*CASB^−^*	Δ*IBB*
+/+	97 ± 1.73	89 ± 4.94	93 ± 4.24	94 ± 2.08
*imp-α2^D14^/+*	20 ± 2.32	76 ± 4.94	86 ± 0.00	87 ± 5.85
*imp-α2^D14^/imp-β^KetRX13^*	2 ± 1.12	0 ± 0.00	90 ± ND	85 ± ND
*imp-α2^D14^/imp-β^KetRP13^*	23 ± 9.19	32 ± 4.04	93 ± ND	91 ± ND
*imp-α2^D14^/imp-β^c02473^*	0 ± 0.00	0 ± 0.00	91 ± ND	87 ± ND
*imp-α2^D14^/imp-β^e02657^*	0 ± 0.00	1 ± 1.12	87 ± ND	83 ± ND
*imp-α2^D14^/imp-β^e03750^*	0 ± 0.00	2 ± 1.76	84 ± ND	79 ± 1.54

On average, 100–300 embryos were scored in each experiment.

± standard deviation; ND, not determined.

a The genotype of second chromosome is given in the first column, and the corresponding mutated domains in the *P{UAS-imp-α2}* transgenes located on the third chromosome are shown in the subheading. All transgenes were expressed by the *nos-Gal4^VP16^* driver on the third chromosome.

### Concurrent reduction in specific *imp-α2* and *imp-β* gene activity blocks mitosis in early embryos

**Figure 1:**
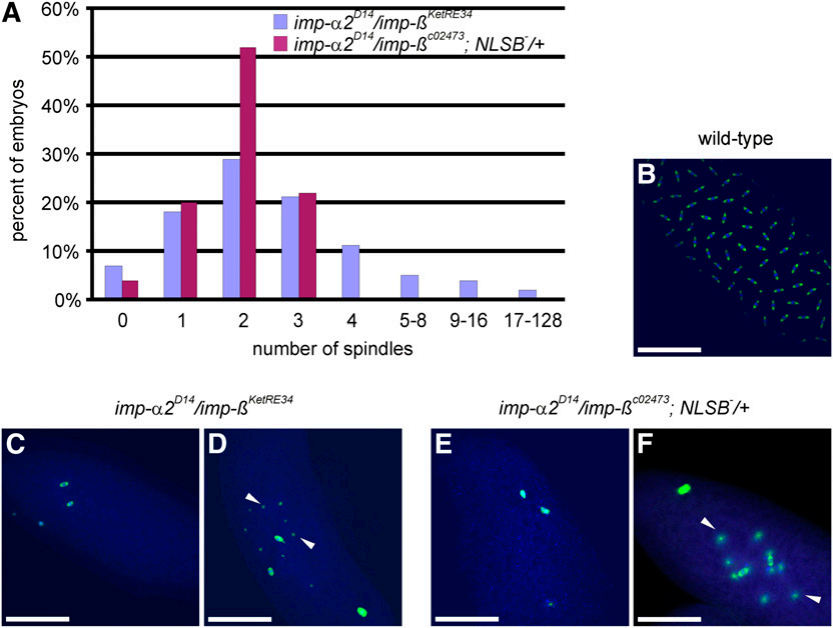
Early embryonic arrest in embryos derived from *imp*-α*2^D14^/imp-β^KetRE34^* and *imp*-α*2^D14^/imp-β^c02473^*; *NLSB^−^/+* females. (A) Mitotic spindle numbers found in 2- to 4-hr-old mutant embryos. At least 100 embryos were scored for each genotype. (B–F) Wild-type and mutant embryos stained for α-tubulin (green) and DNA (blue). (B) Wild-type embryo in the 7^th^ mitotic cycle fixed one hour after egg laying (AEL). (C, D) Mutant embryos derived from *imp-α2^D14^/imp-β^KetRE34^* and (E, F) *imp-α2^D14^/imp-β^c02743^; NLSB^−^/+* females. Arrowheads point to free asters. Scale bar: 100 μm.

**Figure 2:**
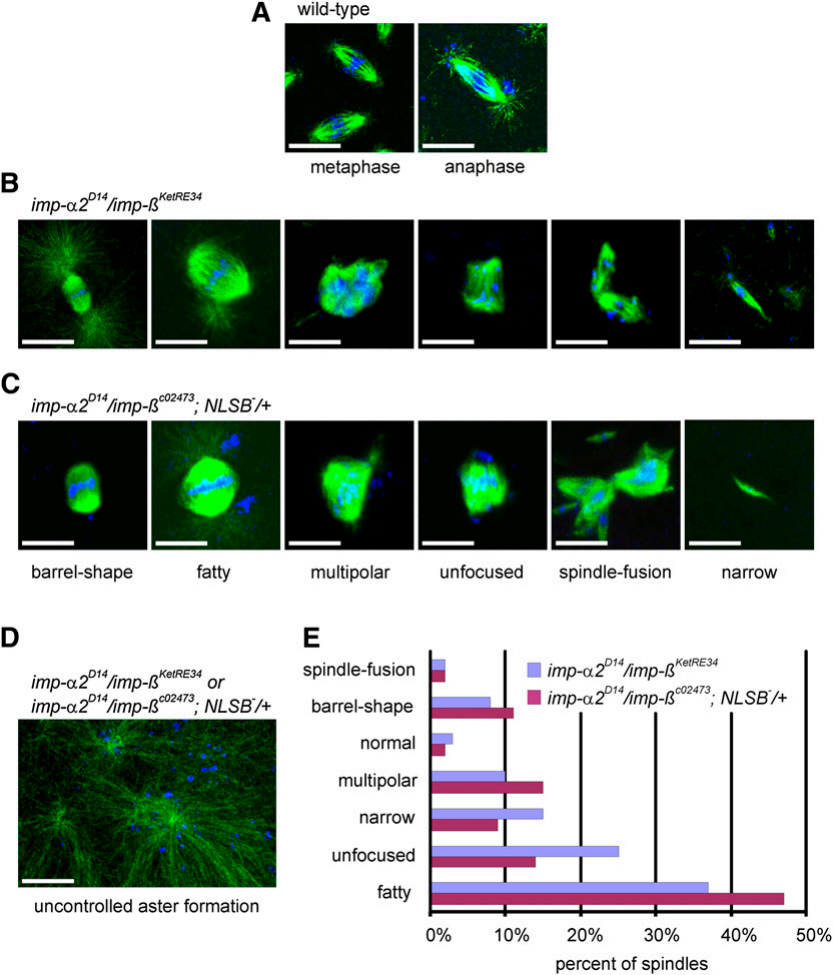
Spindle abnormalities in embryos derived from *imp*-α*2^D14^/imp-β^KetRE34^* and *imp*-α*2^D14^/imp-β^c02473^*; *NLSB^−^/+* females. (A–D) Wild-type and mutant embryos stained for α-tubulin (green) and DNA (blue). (A) Mitotic spindles in wild-type embryos at metaphase and anaphase. (B, C) Categories of spindle abnormalities found in embryos derived from (B) *imp-α2^D14^/imp-β^KetRE34^* and (C) *imp-α2^D14^/imp-β^c02743^*; *NLSB^−^/+* females. (D) Formation of aster networks found in both genotypes. Scale bar: 10 μm. (E) Frequency of spindle defects in embryos from both types of mutant females. Female genotypes are displayed at the upper right corner. At least 200 spindles were scored for both genotypes.

To determine more precisely when the developmental arrest takes place in eggs laid by *imp-α2^D14^/imp-β^KetRE34^* *trans*-heterozygous females or *imp-α2^D14^/imp-β^c02473^*; *nos-Gal4^VP16^*, *P{UAS-imp-α2^NLSB^*^−^*}/+* females (thereafter, *NLSB*^−^ denotes *nos-Gal4^VP16^*, *P{UAS-imp-α2^NLSB^}*), we collected eggs for 2 hr, and aged them for 2 hr before fixation. The embryos were then stained to visualize α-tubulin and DNA. Their development was predominantly blocked during the very first mitotic divisions in cycle 1 to 3 (Figure 1A). As a control, a 1-hr-old wild-type embryo is shown (Figure 1B). In the mutant embryos, all identified nuclei consisted of metaphase-like structures with chromatin in the center and enlarged masses of microtubules organized at both poles (Figure 1, C–F). Centrosomes were variable in number and organization. In addition, arrays of regularly spaced centrosomal structures (Figure 1, D and F) indicate that the cycle of centrosome replication was less hampered than the mitotic cycle, a characteristic also observed in other mutations affecting mitosis (Belecz *et al.* 2001; Zhang *et al.* 2009). Both types of mutant embryos displayed a wide range of abnormalities, among which the formation of free asters was one of the most frequent phenotypes (Figure 1, D and F) detected in about three-quarters of the laid eggs. We found also embryos with microtubules filling the ooplasm and giving rise to a cobweb of free asters (Figure 2D and Figure S2, B and C). Some embryos contained up to 50–60 free asters.

The importance of the cooperation between *imp-α2* and *imp-β* was confirmed by comparing the number of mitotic figures between arrested embryos, including those derived from *imp-α2^D14^/+*; *NLSB^−^/+* or *imp-β^KetRE34^/+* single mutant females (80% and 39% lethality, respectively), and the embryos laid by *imp-α2^D14^/imp-β^c02473^*; *NLSB^−^/+* or *imp-α2^D14^/imp-β^KetRE34^* females (100% lethality). Whereas 95–100% of the embryos from double mutants exhibited fewer than 16 spindles, 35–45% of the lethal embryos from the single mutants displayed from 129 to 4,000 spindles in the ooplasm (Figure S3). Moreover, about 15% of 4- to 6-hr-old arrested embryos from the single mutants were able to partially cellularize, but their development was blocked due to the accumulation of mitotic defects. Our data further point out that embryonic development was arrested significantly earlier in eggs derived from females carrying specific mutations in both *imp-α2* and *imp-β* genes than in those derived from females heterozygous for only one of them, emphasizing the synergy taking place between these two genes.

### Mitotic progression requires interaction between Imp-α2 and Imp-β

Examination by confocal microscopy of embryos derived from *imp-α2^D14^/imp-β^KetRE34^* and *imp-α2^D14^/imp-β^c02473^; NLSB^−^/+* females revealed numerous mitotic defects, which are characterized by a mitotic block during the early nuclear divisions (Figure 2). Essentially no interphase nucleus could be detected in these embryos. In general, the majority of the mitotic figures consisted of considerably enlarged spindles. The most frequently detected type of mitotic abnormality was the occurrence of fatty spindles made of large masses of microtubules originating from both poles and widening out at the equator. In these structures, the chromatin was aligned at the equator, forming a metaphase plate. The barrel-shape spindles, which were smaller than the fatty spindles, might be a form of fatty spindle at the beginning of its growth. We also observed multipolar spindles containing discrete aggregates of chromatin and unfocused spindles in which the chromatin was fragmented in small aggregates. The multipolar spindles and the partially fused spindles at one of the poles might represent remnants of incompletely divided nuclei or might result from replicated centrosomes (*vide infra*). Furthermore, we detected narrow spindles, which were present in about 15% of the mutant embryos. These spindles contained significantly smaller amounts of microtubules, as well as reduced or indetectable amounts of chromatin.

To further characterize the mitotic arrest, we examined the distribution of phospho-histone staining in chromatin and found that, by comparison to wild-type mitosis in which the level of phospho-histone staining was high in condensed chromatin before chromatid separation and lower after chromatid separation (Figure S4, A and B), the level of staining in the mutant embryos was relatively high and equally homogenous in the chromatin aligned on the metaphase plate in fatty spindles (Figure S4C), as well as on the dispersed chromatin spots detected in multipolar spindles (Figure S4D). In contrast, we frequently observed DNA aggregates negatively stained for phospho-histone at the periphery of the spindles (data not shown) or in narrow spindles, which might contain a reduced number of chromatids (Figure S4E). These data indicate that the process of chromatin condensation occasionally becomes dysregulated in the mutant embryos.

**Figure 3:**
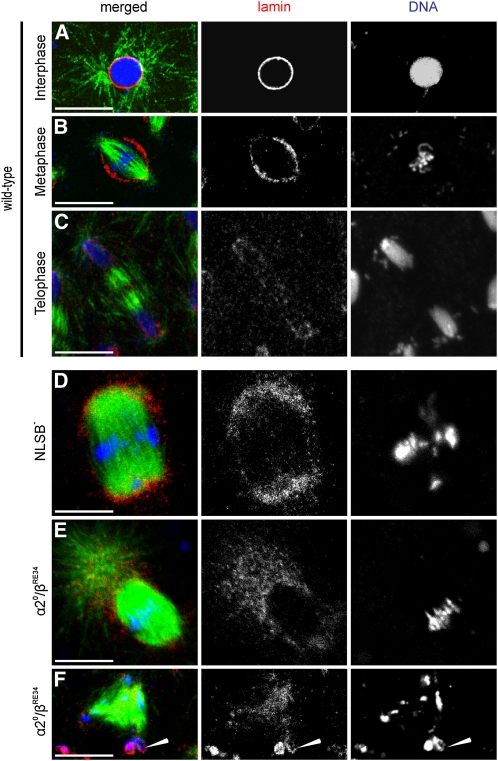
Nuclear envelope organization in embryos derived from *imp-α2^D14^/imp-β^KetRE34^* (α2^0^/β^RE34^) and *imp-α2^D14^/imp-β^c02473^*; *NLSB^−^/+* (*NLSB^−^*) females. Embryos were stained for α-tubulin (green), lamin Dm0 (red), and DNA (blue). (A–C) Wild-type nuclei in (A) interphase, (B) metaphase, and (C) telophase. (D) NLSB^−^ fatty spindle with lamin-aggregates capping both spindle poles. (E) α2^0^/β^RE34^ fatty spindle with lamin vesicles concentrated at the pole covered by a large aster. (F) α2^0^/β^RE34^ multipolar spindle with two chromatin aggregates (arrowhead) located at one of the poles and surrounded by lamin. Scale bar: 10μm.

Similarly, we examined the fate of the nuclear envelope in arrested mitosis of mutant embryos laid by *imp-α2^D14^/imp-β^KetRE34^* and *imp-α2^D14^/imp-β^c02473^*; *NLSB^−^/+* mothers (Figure 3). In mitosis taking place in wild-type embryos, the staining of the nuclear envelope with anti-lamin Dm0 antibodies showed that the interphase nucleus was surrounded by a continuous membrane (Figure 3A), which became broken at the onset of mitosis over both spindle poles. During metaphase, the lamin staining decorated remnants of the nuclear envelope forming a wide belt around the nucleus equator (Figure 3B). In telophase, the nuclear envelope reassembled on the surface of each group of separated chromatids, and the lamin staining capped the forming nuclei (Figure 3C).

In embryos laid by both types of mutant females, we found a distinct pattern of lamin staining. In the fatty spindles with a conspicuous metaphase plate, we found a high concentration of positively stained lamin dots or membrane vesicles capping both poles of the spindle. The lamin-stained vesicles were nearly absent from the equator belt (Figure 3D). The vesicles were detected in a relatively high concentration at the spindle pole harboring a large aster (Figure 3E). In a multipolar spindle, we found that the lamin staining formed an apparently continuous stratum around the chromatin located at one of the poles (Figure 3F), suggesting that a nuclear envelope could be formed when chromatids were pulled from the metaphase plate to one of the spindle poles. Furthermore, we detected less frequently large masses of DNA aggregates encapsulated by an apparently continuous layer of lamin, whose thickness was particularly large (Figure S5, A and A1). We also observed embryos filled with relatively large, positively stained lamin vesicles essentially devoid of DNA (Figure S5, B and B1). These data indicate that a concomitant decrease in *imp-α2* and *imp-β* gene activity resulted in the fragmentation of the nuclear envelope into small vesicles, which essentially accumulated at the spindle poles. Occasionally they were able to reform a nuclear envelope around aggregated chromatids, or they constituted large vesicles predominantly devoid of chromatin.

### Imp-α2/Imp-β complex contributes to centrosome dynamics independently from spindle formation

**Figure 4:**
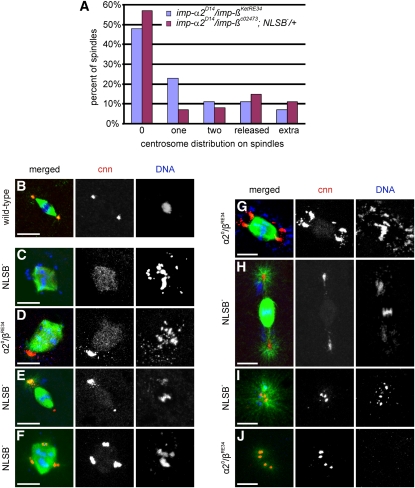
Centrosome organization in embryos derived from *imp-α2^D14^/imp-β^KetRE34^* (*α2^0^/β^RE34^*) and *imp-α2^D14^/imp-β^c02473^*; *NLSB^−^/+* (*NLSB^−^*) females. (A) Number of centrosomes per spindle in eggs laid by mutant females. (B–J) Mitotic figures of embryos from (B) wild-type and (C–J) mutant females stained for α-tubulin (green), centrosomin (cnn, red), and DNA (blue). (C) Centrosomeless spindle. (D) Spindle with one abnormally large centrosome. (E) Barrel-shape spindle with one nearly normal centrosome and one abnormally large detaching centrosome. (F) Multipolar spindle with duplicated centrosomes at each pole. (G) Barrel-shape spindle with two abnormally large centrosomes at each pole of the spindle. (H) Spindle linked to two detached centrosomes associated with aster-forming microtubules. (I, J) Groups of centrosomes located in the cytoplasm and associated with microtubules. Scale bar: 10 μm.

To determine the organization of the centrosome, we examined the distribution of centrosomin, a pericentriolar component (Heuer *et al.* 1995), in wild-type and mutant embryos. We found that the majority of the mutant spindles contained either no centrosome or a single one (Figure 4A), independently of the spindle shape (Figure 4, C and D). In addition, we frequently observed relatively large structures positively stained for centrosomin at one pole (Figure 4, D and E) or at several spots at the periphery of the spindles (Figure 4, F and G). We also detected centrosomes that were detached from the poles, albeit still linked to the spindle through thin microtubules (Figure 4, E and H). Finally, multiple enlarged centrosomal structures could be found independent of large spindles (Figure 4, I and J), and they were associated with astral microtubules or barely detectable microtubules.

The occurrence of supernumerary centrosomal structures associated with one spindle was significant, reaching 7% of all spindles in the embryos derived from *imp-α2^D14^/imp-β^KetRE34^* and 11% in the embryos from *imp-α2^D14^/imp-β^c02473^*; *NLSB^−^/+* females (Figure 4, A, F, and G). We speculate that, by comparison to wild-type, the strongly centrosomine-stained structures detected in Figure 4, D–F may represent aggregates of duplicated centrioles, either unable to move apart (Figure 4, D and E) or separated in an earlier event but then replicating without disjunction (Figure 4, F and G). Alternatively, these aggregates may represent extra-accumulation of pericentriolar material without centriole replication. Our findings indicate that the centrosomes frequently replicate independently of the nuclear cycle, irrespective of whether they are associated with or free from spindles, a phenotype also reported for other mutants (Belecz *et al.* 2001; Zhang *et al.* 2009).

### Imp-α2 forms complexes with ISWI, CP190, and lamin through the NLSB domain

**Figure 5:**
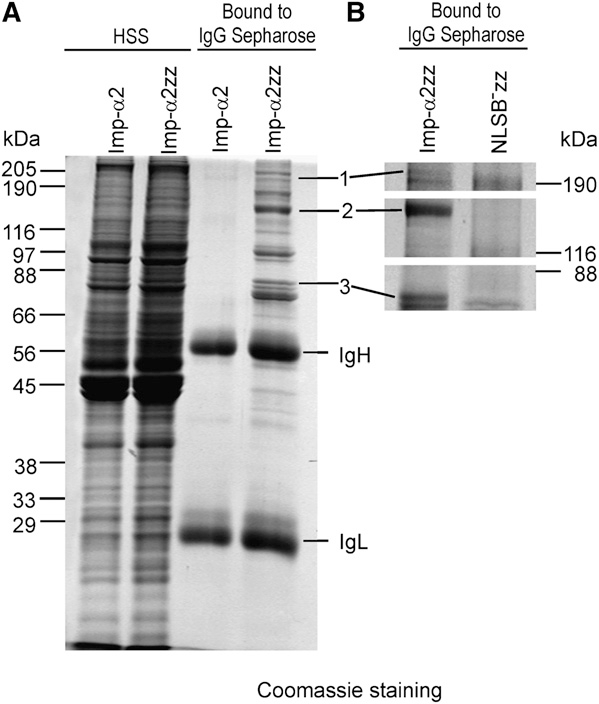
Isolation of *Drosophila* ovarian proteins specifically associated with the NLSB domain of Imp-α2. Proteins were extracted from ovaries of transformed flies producing zz-tagged Imp-α2 (Imp-α2zz) or zz-tagged NLSB^−^ Imp-α2 (NLSB^−^zz). (A) SDS-polyacrylamide gel stained with Coomassie Blue for proteins from high-speed supernatants (HSS) of control and Imp-α2zz extracts (left two lanes). The proteins show equal distribution in both extracts. The HSS proteins were then adsorbed on IgG Sepharose beads, and the eluted proteins were separated on SDS-polyacrylamide gel (right two lanes). (B) The procedure was repeated for Imp-α2zz and NLSB^−^zz ovarian extracts. Protein bands present in the Imp-α2zz purified fraction but absent from the NLSB^−^zz fraction were excised, digested with trypsin and subjected to mass spectrometry. The following proteins were identified in the selected bands: (1) CP190, (2) ISWI, and (3) lamin Dm0.

As mutations in the NLSB domain of *imp-α2* resulted in a strong embryonic phenotype in an *imp-α2^D14^/imp-β^c02473^* background, we performed an analysis to identify partner proteins that would specifically bind to the NLSB domain of Imp-α2. For this purpose, we overexpressed wild-type and NLSB^−^ zz-Imp-α2–tagged proteins (Rigaut *et al.* 1999) in fly ovaries, purified the Imp-α2 complexes by affinity chromatography, eluted the bound proteins, and separated them by SDS-PAGE. In this way, we isolated a relatively large series of protein bands, which were specifically recovered in association with the wild-type zz-Imp-α2 protein, albeit absent among the NLSB^−^ zz-Imp-α2 complexes (Figure 5). These protein bands were excised from the gel, submitted to trypsin digestion, and the nature of the peptides was identified by mass spectrometry. Among the proteins specifically interacting with the NLSB domain of Imp-α2, we identified three protein factors known to be involved in mitosis, which include the ISWI protein acting in spindle assembly, (Yokoyama *et al.* 2009), CP190 involved in centrosome formation (Oegema *et al.* 1995), and lamin Dm0 associated with the nuclear envelope (Lenz-Böhme *et al.* 1997). These data indicate that the *Drosophila* Imp-α2 protein can bind through its NLSB domain with specific factors regulating mitosis.

### Both Imp-β^KetD^ and Imp-β^KetRE34^ proteins bind RanGTP and RanGDP with high affinity

**Figure 6:**
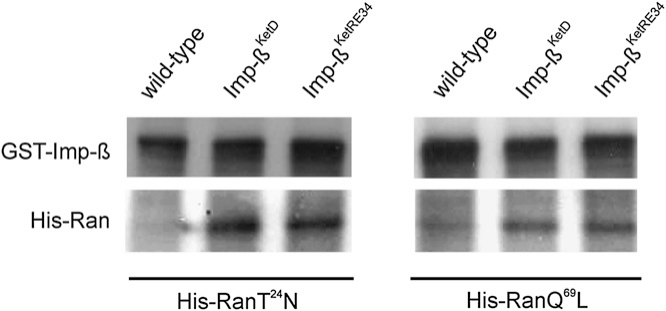
Imp-β^KetD^ and Imp-β^KetRE34^ bind RanGDP and RanGTP with a higher affinity than wild-type Imp-β. His-RanT^24^N (left panel) and His-RanQ^69^L (right panel) proteins, representing the GDP- and GTP-bound forms, respectively, were expressed in bacteria, purified, and subsequently added to wild-type embryonic protein extract. Aliquots of both mixtures were incubated with GST-Imp-β, and either GST-Imp-β^KetD^ or GST-Imp-β^KetRE34^ fusion proteins immobilized on Glutathione Sepharose beads. Proteins bound to the beads were analyzed by SDS-PAGE and immune-detected on Western blot with anti-Ran and anti-Imp-β antibodies.

The mitotic arrest occurring in *imp-α2^D14^/imp-β^c02473^; NLSB^−^/+* could be explained by the dominant toxic effect of the NLSB^−^ construct being unable to bind factors promoting spindle assembly. However, the *imp-β^KetRE34^* deleterious effect could not be directly attributed to Imp-α2 but to modifications occurring in Imp-β that would affect the binding of factors to Imp-α2. The marked enlargement of spindles in mitotically arrested embryos derived from *imp-α2^D14^/imp-β^KetRE34^* females suggests an abnormally high level of active factors involved in spindle assembly. This may indicate an alteration of RanGTP/GDP affinity for the Imp-β^KetRE34^ protein, similar to that found in the original Imp-β^KetD^ mutant protein (Timinszky *et al.* 2002). Therefore, we examined the affinity of Imp-β^KetRE34^ for mutant Ran proteins His-RanQ^69^L and His-RanT^24^N locked in the GTP- and the GDP-bound forms, respectively (Klebe *et al.* 1995). For this purpose, we performed a GST-pulldown experiment with Imp-β, and mutant Imp-β^KetD^ or Imp-β^KetRE34^ proteins. The full-length coding domain of the corresponding cDNAs were cloned in frame with a GST sequence of the *pGEX4-T-2* expression vector. The bacterially synthesized fusion proteins were purified on Glutathione Sepharose beads and mixed with *Xenopus* His-RanT^24^N or His-RanQ^69^L proteins, as well as crude proteins extracted from 0- to 120-min-old *Drosophila* embryos. After washes with binding buffer, the bound Ran proteins were separated by SDS-PAGE and detected by Western blotting with anti-Ran antibodies. As shown on Figure 6 (left panel), the Imp-β^KetRE34^ and Imp-β^KetD^ proteins were able to bind higher amounts of RanGDP than the wild-type Imp-β. Similarly, Imp-β^KetRE34^ and Imp-β^KetD^ displayed a higher binding affinity for RanGTP than Imp-β (right panel). These data are in contrast with previous results showing that Imp-β^KetD^ might have a weaker affinity toward RanGTP than Imp-β (Timinszky *et al.* 2002), but as is shown in the upper panels, we used equal amounts of Imp-β proteins in the reaction mix. The high affinity of RanGTP and RanGDP for Imp-β^KetD^ and Imp-β^KetRE34^ suggests that both mutant proteins could be prone to dissociate from Imp-α2, thus inducing a release of the cargo proteins carried by Imp-α2, or it may prevent their binding to Imp-α2. Consequently, the activity of factors involved in spindle assembly may be permanently enhanced, resulting in the formation of enlarged spindles and a metaphase arrest.

### Reduction in the level of RanGTP can restore viability of *imp-β^KetRE34^* eggs

Because a higher RanGTP/GDP affinity for Imp-β^KerRE34^ may favor its dissociation from Imp-α2 and thus the activation of spindle assembly factors, we tested whether mutations affecting the level of RanGTP/GDP might modify the semidominant lethality in *imp-β^KetRE34^*. As shown in Table 6, the viability of eggs laid by *imp-β^KetRE34^/+* females reached 61%. When we combined *imp-β^KetRE34^* with *Df(3L)w5.4*, which uncovers the *Bj1/RCC1* sequence encoding the Ran guanosine exchange factor (RanGEF), viability of eggs laid by *imp-β^KetRE34^/+*; *Df(3L)w5.4/+* females was strongly enhanced, reaching 96%. When we combined *imp-β^KetRE34^* with loss-of-function mutations in *RanGap*, which encodes the RanGTPase-activating protein, we reduced the viability of the eggs laid by *imp-β^KetRE34^/RanGap^EP1173^* or *imp-β^KetRE34^/RanGap^EY21763^* females to 27% and 28%, respectively. These data showed that a reduction in the level of RanGTP was able to compensate the *imp-β^KetRE34^* mutation, presumably by allowing a functional interaction between Imp-α2 and Imp-β. In contrast, when RanGTP level was increased by reducing the GTPase activity of *RanGap*, the dysfunction of the Imp-β^KetRE34^ protein was enhanced. Alone, the *RanGAP^EP1173^* or *RanGAP^EY21763^* mutation in heterozygous conditions exert no significant decrease in embryo viability.

**Table 6:** Mutations affecting the RanGTP concentration modify the *imp-β^KetRE34^* phenotype

Female Genotype	Egg Viability (%)	SD	*n*
*imp-β^KetRE34^/*+	61	9.50	234
*imp-β^KetRE34^/+; Df(3L)w5.4/+*	96	2.33	310
*imp-β^KetRE34^/RanGap^EP1173^*	27	6.55	728
*imp-β^KetRE34^/RanGap^EY21763^*	28	4.72	395
*RanGap^EP1173^*/+	92	6.92	150
*RanGap^EY21763^/+*	81	3.05	150

Deletion *Df(3L)w5.4* uncovers *Bj1/RCC1* gene (encoding RanGEF).

*n*, number of embryos scored.

## Discussion

This study reveals that *Drosophila* *imp-α2* plays a specific role in early embryogenesis and cannot be substituted with *imp-α1* or *imp-α3*. Furthermore, the cooperation between *imp-α2* and *imp-β* is strictly required to regulate the organization of microtubules, centrosomes, and the nuclear envelope throughout mitosis in preblastoderm-stage *Drosophila* embryos.

### Balanced cooperation between Imp-α2 and Imp-β is essential to enable rapid synchronous nuclear divisions in the *Drosophila* syncytial embryo

The Ran-regulated cooperation between Imp-α2 and Imp-β controls the activity of a variety of NLS-containing SAF proteins. Imp-α2 and Imp-β are synthesized during oogenesis, stored in relatively large amounts in *Drosophila* and *Xenopus* eggs (Görlich *et al.* 1994; Gruss *et al.* 2001; Jans *et al.* 2000). During the first 2 hr of *Drosophila* embryogenesis, essentially no zygotic gene expression takes place and the rapid synchronous divisions within the syncytial cytoplasm are exclusively driven by maternally deposited gene products (Foe *et al.* 1993). Even when the amount is reduced by half the maternal input of Imp-α2 and Imp-β proteins is sufficient to drive mitotic divisions.

The findings of a genetic interaction between, on the one hand, *imp-α2^D14^* and *imp-β^KetRE34^* and, on the other hand, *imp-α2^NLSB^*^−^ or *imp-α2^SNLSB^*^−^ and null alleles of *imp-β* suggest that a critical threshold of functional Imp-α2/Imp-β complexes should be maintained to sustain mitosis during early embryogenesis. One gene dosage of *imp-α2^NLSB^*^−^ or *imp-α2^SNLSB^*^−^ alleles reduced the amount of functional complexes, while *imp-β^KetRE34^*, as inferred from pulldown experiments and mitotic phenotype of the genetic interactions, appears to decrease the stability of the NLS-protein/Imp-α2/Imp-β ternary complex. In embryos from *imp-α2^D14^/imp-β^KetRE34^* transheterozygous females, the Imp-β^KetRE34^ and wild-type Imp-β proteins display a 1:1 ratio (data not shown) and compete for a reduced amount of Imp-α2 (50% of normal level). Analysis the docking of the IBB domain of Imp-α2 on Imp-β^KetRE34^ and wild-type Imp-β by computer modeling revealed no striking difference in their binding affinities for the IBB domain. This result supports the assumption that the binding affinity of Imp-β^KetRE34^ or wild-type Imp-β for a full-length Imp-α2 is similar. Therefore, the amount of functional Imp-β and Imp-α2 complexes in embryos laid by *imp-α2^D14^/imp-β^KetRE34^* females should be reduced to a subthreshold level, inadequate to regulate early embryonic mitosis.

An analogous argumentation could be used for explaining the lethality of embryos expressing *imp-α2^NLSB^*^−^ or *imp-α2^SNLSB^*^−^ in an *imp-α2^D14^/imp-β^0^* background. In these embryos the mutations affecting the NLS-binding domain of Imp-α2 dominantly blocked the binding of specific cargos to the Imp-α2/Imp-β complexes but should not prevent the formation of these complexes, as the IBB domain remains intact. It is also possible that NLS-mutated Imp-α2 proteins through their intact IBB domain induced a preferential binding to the NLS-domain of intact Imp-α2 proteins and thus prevented the binding of cargos to the Imp-α2/Imp-β complex, resulting in a 100% arrest of embryonic development.

All these data point out the importance of the NLSB domain in the regulation of the rapid nuclear divisions taking place in syncytial embryos and suggest that critical SAF proteins bind to the Imp-α2/Imp-β complex through their NLS sequences. Moreover our finding that SAF are specifically recovered in association with the NLS-binding domain of Imp-α2 further strengths our assumption.

### Molecular nature of the mutant Imp-β proteins

Compared with the original *imp-β^KetD^* allele, we found that *imp-β^KetRE34^* contains a second site mutation (substitution D^725^N) potentially responsible for the partial suppression of the dominant female sterile phenotype of *imp-β^KetD^*. However, it is possible that mutations reducing the expression of the dominant negative Imp-β^KetD^ protein (*e.g.* mutations in the promoter or the UTRs) could also weaken the dominant negative phenotype, but the occurrence of a third site mutation could be considered negligible.

Furthermore, the intragenic mutation resulting in S^317^T substitution, which fully suppresses the dominance of *imp-β^KetD^* (Timinszky *et al.* 2002) indicates that a second site substitution could be sufficient to change the neomorphic function of *imp-β^KetD^*.

### Specific role of Imp-α2

Each of the three Imp-α proteins displays distinct, but partly overlapping, roles in development (Hogarth *et al.* 2006; Mason and Goldfarb 2009). These specific Imp-α functions may be driven during the evolution of Metazoan by unique requirements in gametogenesis (Geles and Adam 2001; Hogarth *et al.* 2006; Mason and Goldfarb 2009). While *Drosophila* homozygous mutants of *imp-α1* and *imp-α2* grow to adulthood, both females and males remain sterile, indicating specific roles of Imp-α1 and Imp-α2 in spermatogenesis (Giarrè *et al.* 2002; Mason *et al.* 2002; Ratan *et al.* 2008) and oogenesis (Gorjánácz *et al.* 2002; Mason *et al.* 2002; Ratan *et al.* 2008), respectively. The *imp*-*α3* null mutant is zygotic lethal (Mason *et al.* 2002; Máthé *et al.* 2000), and although it is also expressed in spermatids, testes and ovaries (Giarrè *et al.* 2002; Hogarth *et al.* 2006; Máthé *et al.* 2000), its function is basically devoted to nuclear transport (Chan *et al.* 2008; Fang *et al.* 2001).

The specific roles of the three Imp-α proteins during early embryogenesis of *Drosophila* have not been examined. The data of the *UTR^Δ^*-*imp-α* experiments clearly show a specific role of Imp-α2 in the syncytial divisions of *Drosophila* embryos. This finding is in accordance with previous data showing that transgenes carrying mutations in the SNLSB and IBB domains of Imp-α2 were able to rescue the dumpless phenotype of *imp-α2^D14^* homozygous females but were unable to sustain embryogenesis of the rescued eggs (Gorjánácz *et al.* 2006). Similar observations were reported in other metazoans ranging from *C. elegans* to human: in cooperation with Imp-β, the Imp-α2 orthologs act as regulators of mitotic spindle assembly (Askjaer *et al.* 2002; Nachury *et al.* 2001; Ribbeck *et al.* 2007; Schatz *et al.* 2003). Our analysis also indicates that Imp-α2 contributes to the regulation of mitosis in the *Drosophila* syncytial embryo.

The requirement for large amounts of Imp-α2 protein in *Drosophila* eggs could be explained by the need of a sufficient supply to regulate the rapid synchronous mitotic events taking place during early embryogenesis that lead to the formation of 6000 nuclei in about 2.5 hr (Foe *et al.* 1993). When the nuclei become cellularized, the following cell divisions occur at a much-reduced pace and can occur in the absence of Imp-α2, leading to the formation of fully viable, albeit sterile adults (Gorjánácz *et al.* 2002; Török *et al.* 1995) that display defects in muscle patterning and organization of the neuromuscular junction (Mosca and Schwarz 2010a, 2010b). In normal eukaryotic cells, some of the factors involved in mitosis are imported to the nucleus to be sequestered from the mitotic apparatus before the breakdown of the nuclear envelope (Kisurina-Evgenieva *et al.* 2004; Raemaekers *et al.* 2003; Walczak and Heald 2008). In *Drosophila* eggs, these “shuttling” proteins are deposited in an amount large enough to form thousands of spindles and should become available for nuclear import according to the number of dividing nuclei. These proteins should be stored inactive in the cytoplasm until they are needed in the nucleus. Binding to Imp-α2 through their NLS sequence could be a plausible solution for this problem. In this respect, the CP190 protein that can be recovered in association with Imp-α2 constitutes a good example, as this protein shuttles between the nucleus and the centrosomes in a cell cycle–specific manner (Kellogg and Alberts 1992; Oegema *et al.* 1995, 1997). Interestingly, no CP190 was found in the nuclei of *Drosophila* cleavage embryo prior to cycle 10, but it was detected in the cytoplasm and at centrosomes (Frasch *et al.* 1986). Moreover CP190 is involved in axial expansion of the nuclei along the anterior-to-posterior axis of the embryo (Chodagam *et al.* 2005). We suppose that prior to cycle 10, the binding of CP190 with Imp-α2 may be responsible for keeping it inactive in the cytoplasm located beyond a certain distance from centrosomes.

### Imp-α2 and Imp-β regulate the mitotic processes in the syncytial embryo

The role of Importin-β in spindle assembly was demonstrated in *Xenopus* egg extract (Nachury *et al.* 2001). Although the *Drosophila* *imp-β* is a well-characterized gene, identified through its dominant *imp-β^KetD^* mutation causing female sterility (Erdélyi *et al.* 1997; Lippai *et al.* 2000), its involvement in spindle assembly has not been clearly shown. The development in the eggs from *imp-β^KetD^/+* females was blocked at the first cleavage division, the gonomeric spindle failed to form, and disorganized masses of microtubules were observed (Tirián *et al.* 2000). However, when purified Imp-β^KetD^ protein was injected into wild-type syncytial *Drosophila* embryos, the NE formation was blocked, but neither spindle nor spindle envelope defects could be detected (Timinszky *et al.* 2002). Although the spindle abnormalities detected in the analysis of *imp-β^KetRE34^* bear no direct relationship with the *imp-β^KetD^* phenotype, it clearly shows the critical role of *imp-β* in the process of spindle formation.

Our data suggest that overgrown spindles could result from the activity of factors that trigger a persistent microtubule formation in the spindle area, because the absence of functional Imp-α2/Imp-β complexes prevents the sequestration of SAFs and further hampers other factors to foster mitotic progression. Therefore, mitosis was predominantly blocked at the metaphase to anaphase transition.

The observed abnormalities of chromosome condensation, alignment, and separation in embryos developing from *imp-α2^D14^/imp-β^KetRE34^* and *imp-α2^D14^/imp-β^c02473^*; *NLSB^−^/+* females could be a secondary consequence of the spindle defects. However, the finding that the fly ortholog of ISWI was coimmunoprecipitated with Imp-α2 in a NLSB domain-dependent manner suggests that altered concentration of free NLS-bearing proteins could also have a direct effect on the above processes in the mutant embryos. This hypothesis is supported by the recent finding that the ISWI protein, a chromatin-remodeling ATPase (Brown *et al.* 2007; Corona *et al.* 1999; Siriaco *et al.* 2009), was also identified as a RanGTP-dependent MAP required for chromosome segregation and anaphase microtubule stabilization in the *Xenopus* egg and *Drosophila* S2 cells (Yokoyama *et al.* 2009).

The lamin proteins associated with the internal side of the NE play a central role in the nuclear organization by binding nuclear membrane components and DNA (Melcer *et al.* 2007). Reducing the level of Imp-α in *C. elegans* embryos (Geles *et al.* 2002) or elevating its concentration in *Xenopus* egg extract (Adam *et al.* 2008) results in lamin mislocalization or lamin B accumulation in distinct patches on the surface of the chromatin. Our finding that lamin forms aggregates in defective Imp-α2/Imp-β embryos points out that *in vivo* the Imp-α2/Imp-β complex prevents lamin to form spontaneous aggregates, an intrinsic property of lamin dimers detected earlier *in vitro* (Moir *et al.* 1991).

Histone H2A-mediated attachment of lamin to chromosomes is an important step in nuclear lamina assembly (Mattout *et al.* 2007). However, in extracts of *Xenopus* egg, the assembly of the NE was observed on RanGTP-coated beads even in the absence of chromatin (Zhang and Clarke 2000). It is not clear how Ran directs NE assembly, but the mechanism involves the Importins (Brittle and Ohkura 2005; Clarke and Zhang 2008). The formation of spherical lamin Dm0 structures without DNA, which were detected in the mutant embryos, indicates that the factors released from Imp-α2/Imp-β complexes could form NE independently of chromatin.

Altogether, the comparison of the normal lamin pattern (Paddy *et al.* 1996; Walker *et al.* 2000; and this study) with that detected in embryos defective for Imp-α2/Imp-β and the finding of the association of lamin Dm0 with Imp-α2 highlight the importance of RanGTP–Imp-α/Imp-β pathway in the organization of NE assembly. In conclusion, our work shows that Imp-α2 and Imp-β specifically cooperate *in vivo* to essentially regulate spindle dynamics and events related to mitosis during the early nuclear divisions in *Drosophila* embryos.

## Supplementary Material

Supporting Information
