# Supplementary material for: Specific Cooperation Between Imp-α2 and Imp-β/Ketel in Spindle Assembly During Drosophila Early Nuclear Divisions
Source: G3 (Bethesda). 2012 Jan 1;2(1):1–14. doi: 10.1534/g3.111.001073 (PMC3276186; doi:10.1534/g3.111.001073)
Supplement: Supporting Information [file supp_2_1_1__index.html]

Supporting Information 

# Specific Cooperation Between Imp-α2 and Imp-β/Ketel in Spindle Assembly During *Drosophila* Early Nuclear Divisions

## Supporting Information for Viragh *et al.*, 2012

**Files in this Data Supplement:**

- Supporting Information - Figures S1-S5 and Tables S1-S3 (PDF, 5.9 MB)
- Figure S1 - Conformation of the Imp�βD725N mutant protein and intramolecular polar interactions formed by the Asn 725 residue (PDF, 268 KB)
- Figure S2 - Overgrowing free asters in 2-4 h old embryos from mutant females (PDF, 3.2 MB)
- Figure S3 - Quantification of spindle numbers in 4�6 h old developmentally arrested embryos derived from mutant females shows synergistic interaction between specific mutant alleles of *imp�α2* and *imp�β* (PDF, 308 KB)
- Figure S4 - Chromosome abnormalities in phospho-histone-stained embryos from *imp�α2D14/imp�βKetRE34 (α20/βRE34)* and *imp�α2D14/imp�βc02743; NLSB-/+* (NLSB-) mutant females (PDF, 1.1 MB)
- Figure S5 - Defects of nuclear envelope assembly in embryos from *imp�α2D14/imp�βKetRE34 (α20/βRE34)* and *imp�α2D14/imp�βc02743; NLSB-/+* (NLSB-) females (PDF, 900 KB)
- Table S1 - RNAi silencing of *imp�α2* in heterozygous *imp�βKetRE34* females strongly reduces egg viability (PDF, 52 KB)
- Table S2 - Effect of D725N substitution on docking energy of the IBB domain of Imp�α2 as a ligand on Imp�β as a receptor (PDF, 48 KB)
- Table S3 - Effects of RNAi silencing of the three *imp-α* genes on embryo viability in combination with *imp�βKetRE34* (PDF, 52 KB)
