# Supplementary material for: Specific Cooperation Between Imp-α2 and Imp-β/Ketel in Spindle Assembly During Drosophila Early Nuclear Divisions
Source: G3 (Bethesda). 2012 Jan 1;2(1):1–14. doi: 10.1534/g3.111.001073 (PMC3276186; doi:10.1534/g3.111.001073)
Supplement: Supporting Information [file supp_2.1.1_001073SI.pdf]

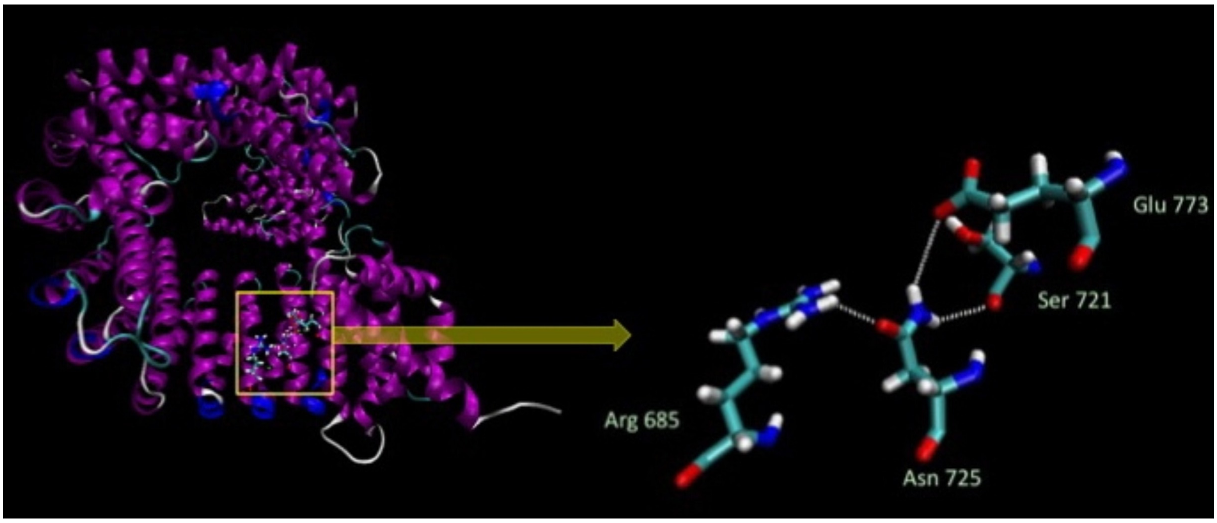

Figure S1 Conformation of the Imp-β^{D725N} mutant protein and intramolecular polar interactions formed by the Asn 725 residue. Interaction between Asn 725 (HEAT repeat 16) and Glu 773 (HEAT repeat 17) stabilizes the relative positions between B helices of HEAT repeat 16 and 17. *In silico* analysis was performed with computer software MODELLER (http://www.salilab.org/modeller/) using the structure of human Importin-β from pdb-database (Code: 1QGK) as template. Energy evaluations were done by the Atomic Non-Local Environment Assessment (ANOLEA**)** program. Secondary structures in overview (left): alpha helix::purple, 3-10 helix::blue, turn::cyan, coil::white. Atoms in detailed view (right): H::white, C::cyan, N::blue, O::red. H-bonds::white dotted lines.

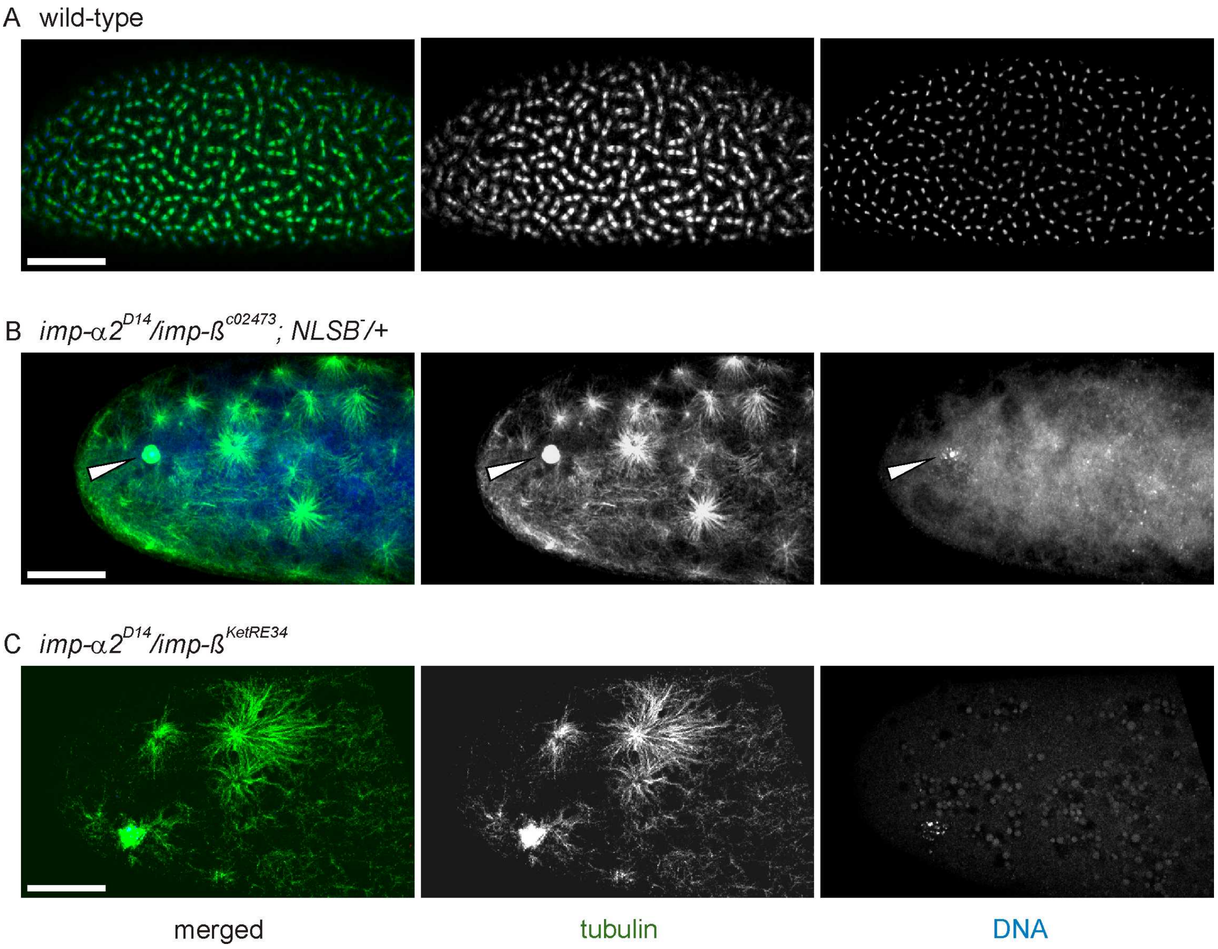

Figure S2 Overgrowing free asters in 2-4 h old embryos from mutant females. (A) Wild-type embryo. (B) Mutant embryo derived from *imp-α2*^{D14}*/imp-β*^{c02743}*; NLSB*$^{-}$/+ females. Arrowhead points to Polar Body. (C) Mutant embryo derived from *imp-α2*^{D14}*/imp-β*^{KetRE34} females. α-tubulin (green) and DNA (blue). Scale bar: 50 µm.

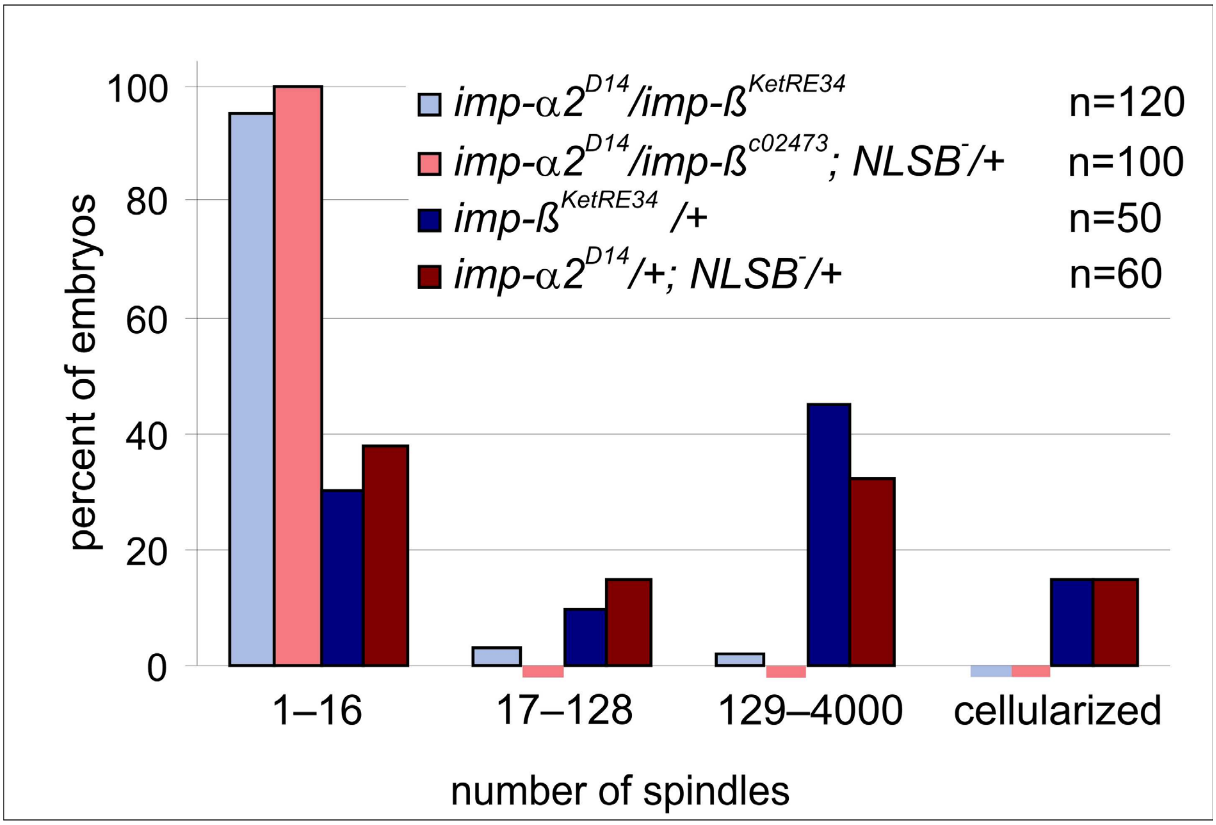

Figure S3 Quantification of spindle numbers in 4-6 h old developmentally arrested embryos derived from mutant females shows synergistic interaction between specific mutant alleles of *imp-α2* and *imp-β*. n=number of embryos scored.

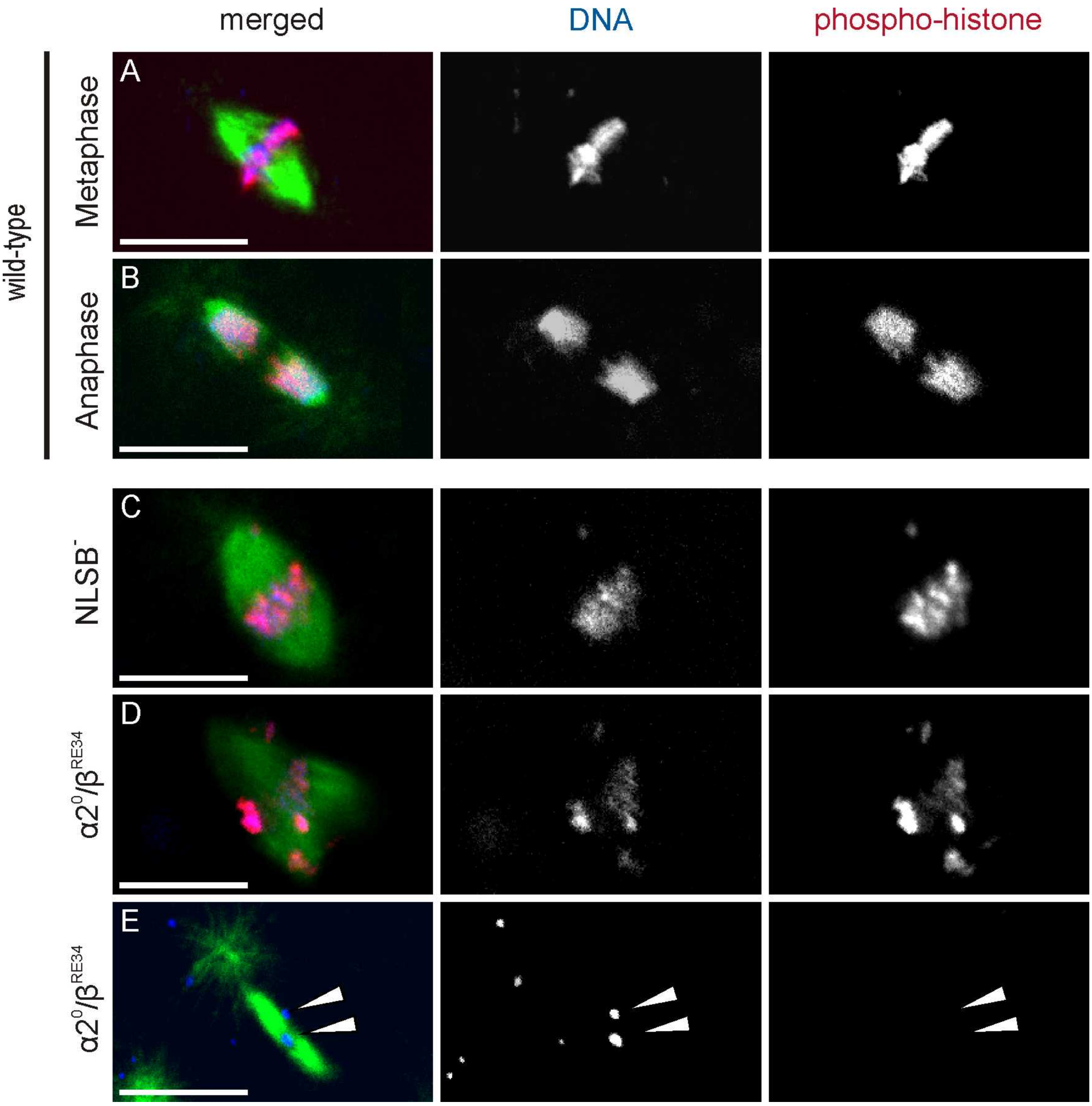

Figure S4 Chromosome abnormalities in phospho-histone-stained embryos from *imp-α2*^{D14}*/imp-β*^{KetRE34} (*α2*^{0}*/β*^{RE34}) and *imp-α2*^{D14}*/imp-β*^{c02743}*; NLSB*$^{-}$*/+* (NLSB^{-}) mutant females. (A, B) Metaphase and anaphase spindles (resp.) in wild-type embryo. (C-E) Spindles in embryos derived from mutant females. (C) Phospho-histone positive, condensed chromosomes aligned at the metaphase-plate of a fatty spindle. (D) Phospho-histone positive chromosomes irregularly scattered on a multipolar spindle. (E) Phospho-histone negative, non-condensed chromatin fragments (arrowheads) on a narrow spindle. α-tubulin (green), phospho-histone (condensed mitotic DNA, red) and DNA (blue). Scale bar: 10µm.

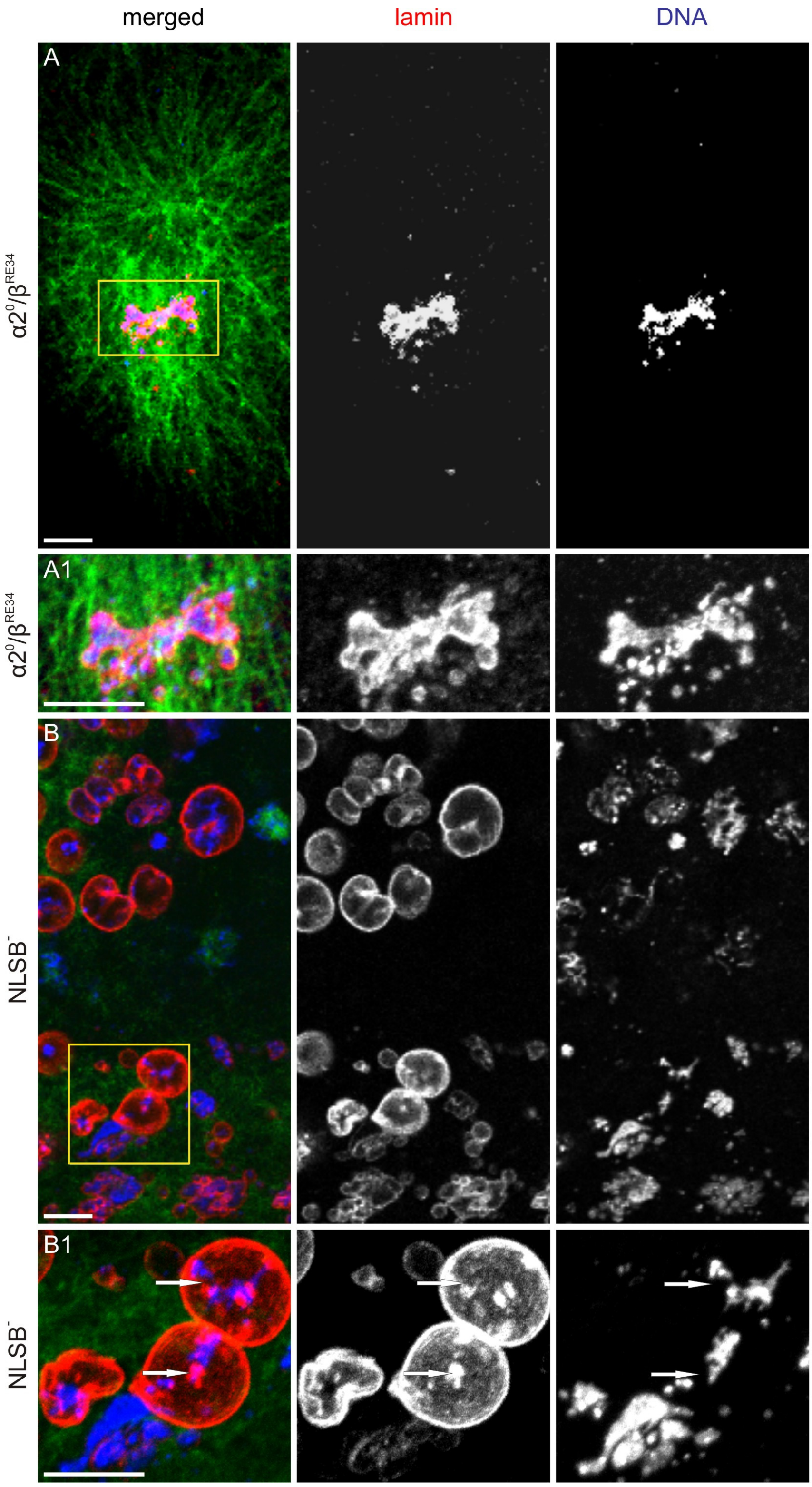

Figure S5 Defects of nuclear envelope assembly in embryos from *imp-α2*^{D14}*/imp-β*^{KetRE34} (*α2*^{0}*/β*^{RE34}) and *imp-α2*^{D14}*/imp-β*^{c02743}*; NLSB*$^{-}$*/+* (NLSB^{-}) females. (A, A1) Chromatin fragments surrounded by lamin on the background of an aster-like accumulation of microtubules. (B, B1) Abnormally large lamin spheres enclosing chromatin fragments and lamin aggregates (arrows). α-tubulin (green), lamin Dm0 (red) and DNA (blue). Scale bar: 10µm.

Table S1 RNAi silencing of *imp-α2* in heterozygous *imp-β*^{KetRE34} females strongly reduces egg viability.

Female genotype	Egg viability (%)	SD	n
imp-α2^{D14}/+	89	2.49	285
imp-β^{KetRE34}/+	61	9.50	234
P{imp-α2i}/ nos-Gal4^{VP16}	93	3.42	150
imp-α2^{D14}/+; *P{imp-α2i}/ nos-Gal4*^{VP16}	57	4,55	176
imp-β^{KetRE34}/+; *P{imp-α2i}/ nos-Gal4*^{VP16}	3	1.73	530

SD=standard deviation, n=number of embryos scored

Table S2 Effect of $D^{725}N$ substitution on docking energy of the IBB domain of Imp-α2 as a ligand on Imp-β as a receptor

Receptor	Ligand[a]	Lowest docking energy [kcal/mole][b]
Imp-β	Imp-α2 IBB domain	-75.63
Imp-β^{D725N}	Imp-α2 IBB domain	-75.53

Molecular structures of wild-type Drosophila Imp-β and the IBB (Importin Beta Binding) domain of wild-type Drosophila Imp-α2 are according to that of detected for human Imp-β complexed with the IBB domain of human Imp-α (PDB – ID: 1QGK). The structure of Drosophila Imp-β^{D725N} is generated by homology modelling using that of the wild type as template.

[a] Residues 17-53 of the IBB domain in helical conformation.
[b] Lowest values of 100 dockings.

Table S3 Effects of RNAi silencing of the three *imp-α* genes on embryo viability in combination with *imp-β*^{KetRE34}

Female genotype	Egg viability (%)	SD	n
imp-β^{KetRE34}/+	61	9.50	234
imp-β^{KetRE34}/ *P{imp-α1i}*; *nos-Gal4*^{VP16}/+	40	5.03	254
imp-β^{KetRE34}/+; *P{imp-α2i*^{34265}*}*/ *nos-Gal4*^{VP16}	0	0.00	610
imp-β^{KetRE34}/+; *P{imp-α2i*^{34266}*}*/ *nos-Gal4*^{VP16}	3	3.07	531
P{imp-α3i^{36104}*}*/+; *imp-β*^{KetRE34}/+; *nos-Gal4*^{VP16}/+	44	2.64	250
imp-β^{KetRE34}/ *P{imp-α3i*^{36103}*}*; *nos-Gal4*^{VP16}/+	43	2.82	123

SD=standard deviation, n=number of embryos scored
