# Supplementary material for: Specific Cooperation Between Imp-α2 and Imp-β/Ketel in Spindle Assembly During Drosophila Early Nuclear Divisions
Source: G3 (Bethesda). 2012 Jan 1;2(1):1–14. doi: 10.1534/g3.111.001073 (PMC3276186; doi:10.1534/g3.111.001073)
Supplement: Supporting Information [file supp_2.1.1_FigureS1.pdf]

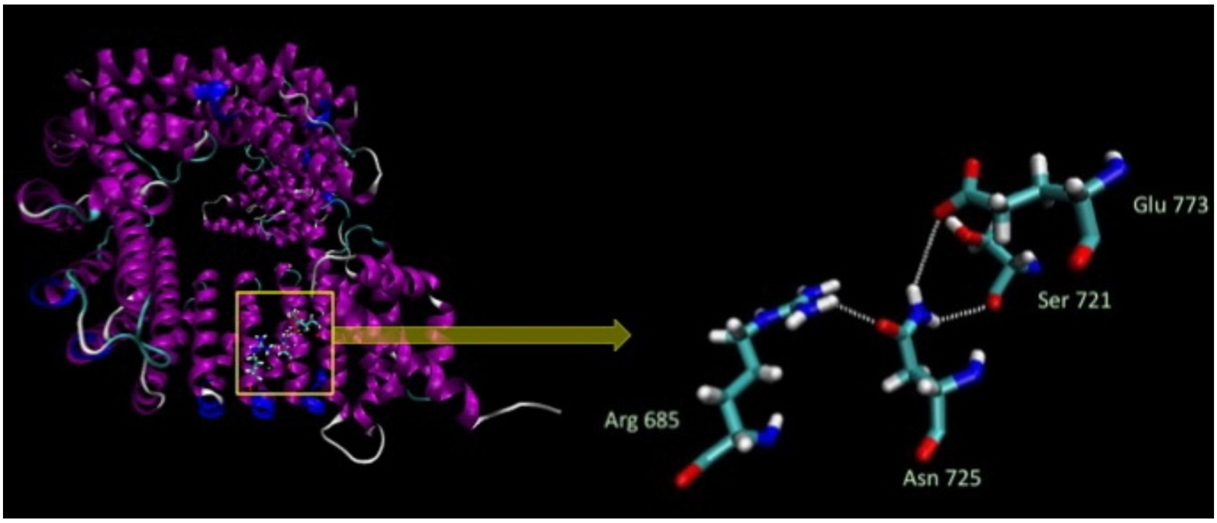

Figure S1 Conformation of the Imp-β^{D725N} mutant protein and intramolecular polar interactions formed by the Asn 725 residue. Interaction between Asn 725 (HEAT repeat 16) and Glu 773 (HEAT repeat 17) stabilizes the relative positions between B helices of HEAT repeat 16 and 17. *In silico* analysis was performed with computer software MODELLER (http://www.salilab.org/modeller/) using the structure of human Importin-β from pdb-database (Code: 1QGK) as template. Energy evaluations were done by the Atomic Non-Local Environment Assessment (ANOLEA**)** program. Secondary structures in overview (left): alpha helix::purple, 3-10 helix::blue, turn::cyan, coil::white. Atoms in detailed view (right): H::white, C::cyan, N::blue, O::red. H-bonds::white dotted lines.
