# Supplementary material for: Specific Cooperation Between Imp-α2 and Imp-β/Ketel in Spindle Assembly During Drosophila Early Nuclear Divisions
Source: G3 (Bethesda). 2012 Jan 1;2(1):1–14. doi: 10.1534/g3.111.001073 (PMC3276186; doi:10.1534/g3.111.001073)
Supplement: Supporting Information [file supp_2.1.1_FigureS2.pdf]

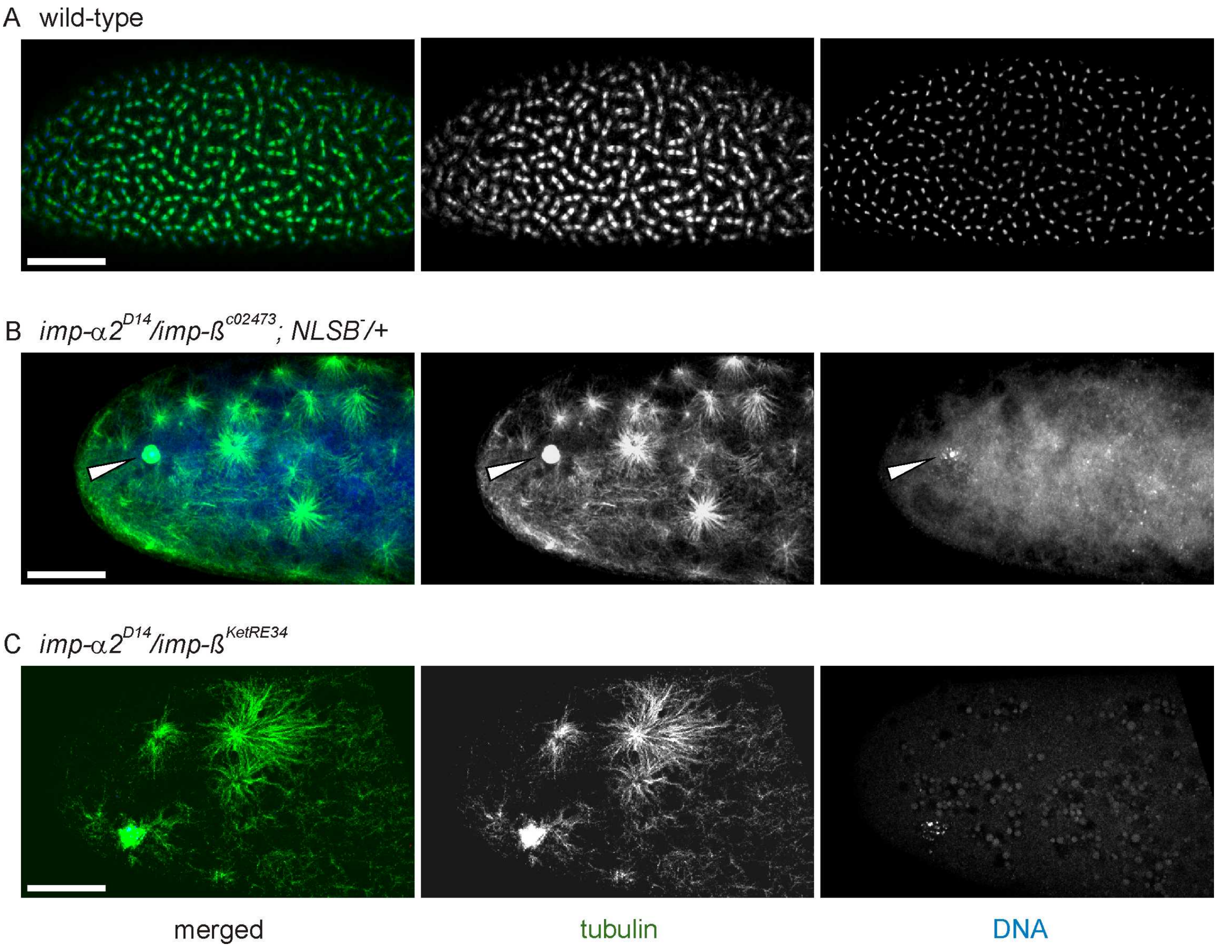

Figure S2 Overgrowing free asters in 2-4 h old embryos from mutant females. (A) Wild-type embryo. (B) Mutant embryo derived from *imp-α2*^{D14}*/imp-β*^{c02743}*; NLSB*$^{-}$/+ females. Arrowhead points to Polar Body. (C) Mutant embryo derived from *imp-α2*^{D14}*/imp-β*^{KetRE34} females. α-tubulin (green) and DNA (blue). Scale bar: 50 µm.
