# Supplementary material for: Specific Cooperation Between Imp-α2 and Imp-β/Ketel in Spindle Assembly During Drosophila Early Nuclear Divisions
Source: G3 (Bethesda). 2012 Jan 1;2(1):1–14. doi: 10.1534/g3.111.001073 (PMC3276186; doi:10.1534/g3.111.001073)
Supplement: Supporting Information [file supp_2.1.1_FigureS3.pdf]

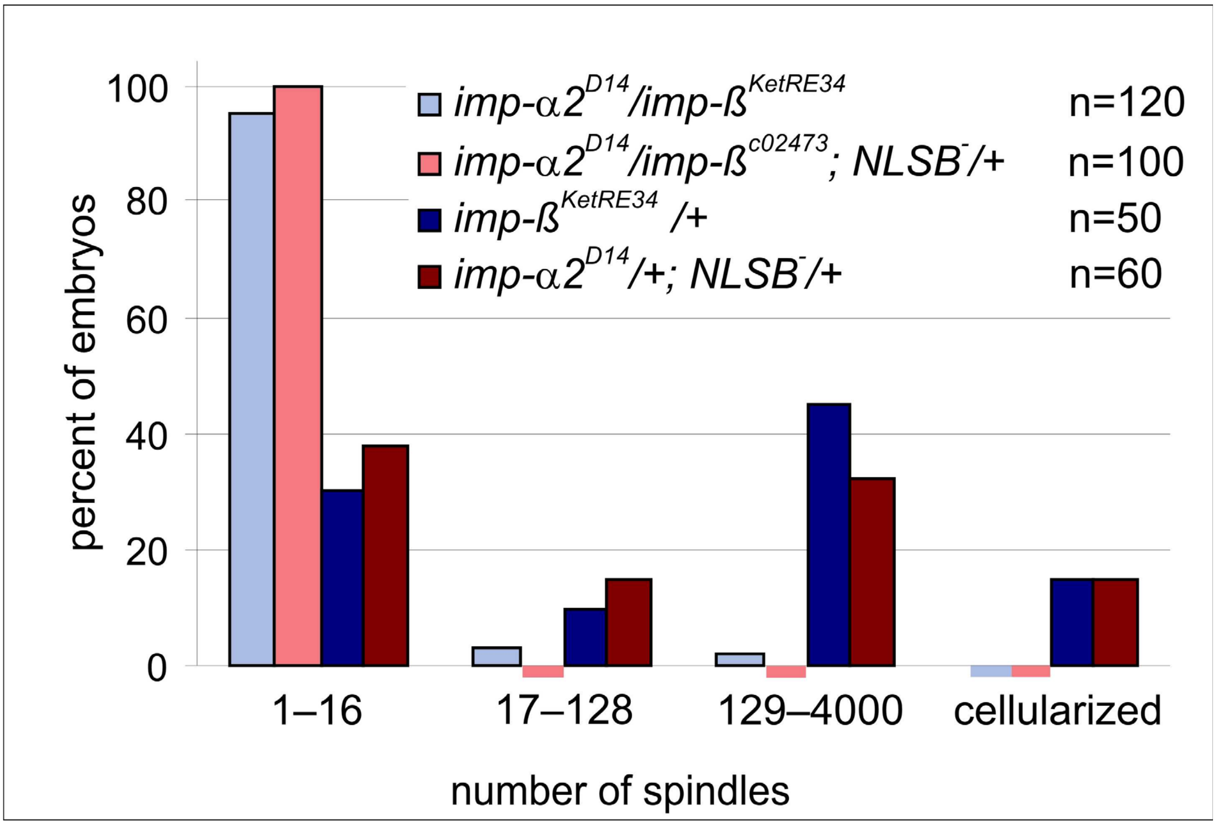

Figure S3 Quantification of spindle numbers in 4-6 h old developmentally arrested embryos derived from mutant females shows synergistic interaction between specific mutant alleles of *imp-α2* and *imp-β*. n=number of embryos scored.
