# Supplementary material for: Specific Cooperation Between Imp-α2 and Imp-β/Ketel in Spindle Assembly During Drosophila Early Nuclear Divisions
Source: G3 (Bethesda). 2012 Jan 1;2(1):1–14. doi: 10.1534/g3.111.001073 (PMC3276186; doi:10.1534/g3.111.001073)
Supplement: Supporting Information [file supp_2.1.1_FigureS4.pdf]

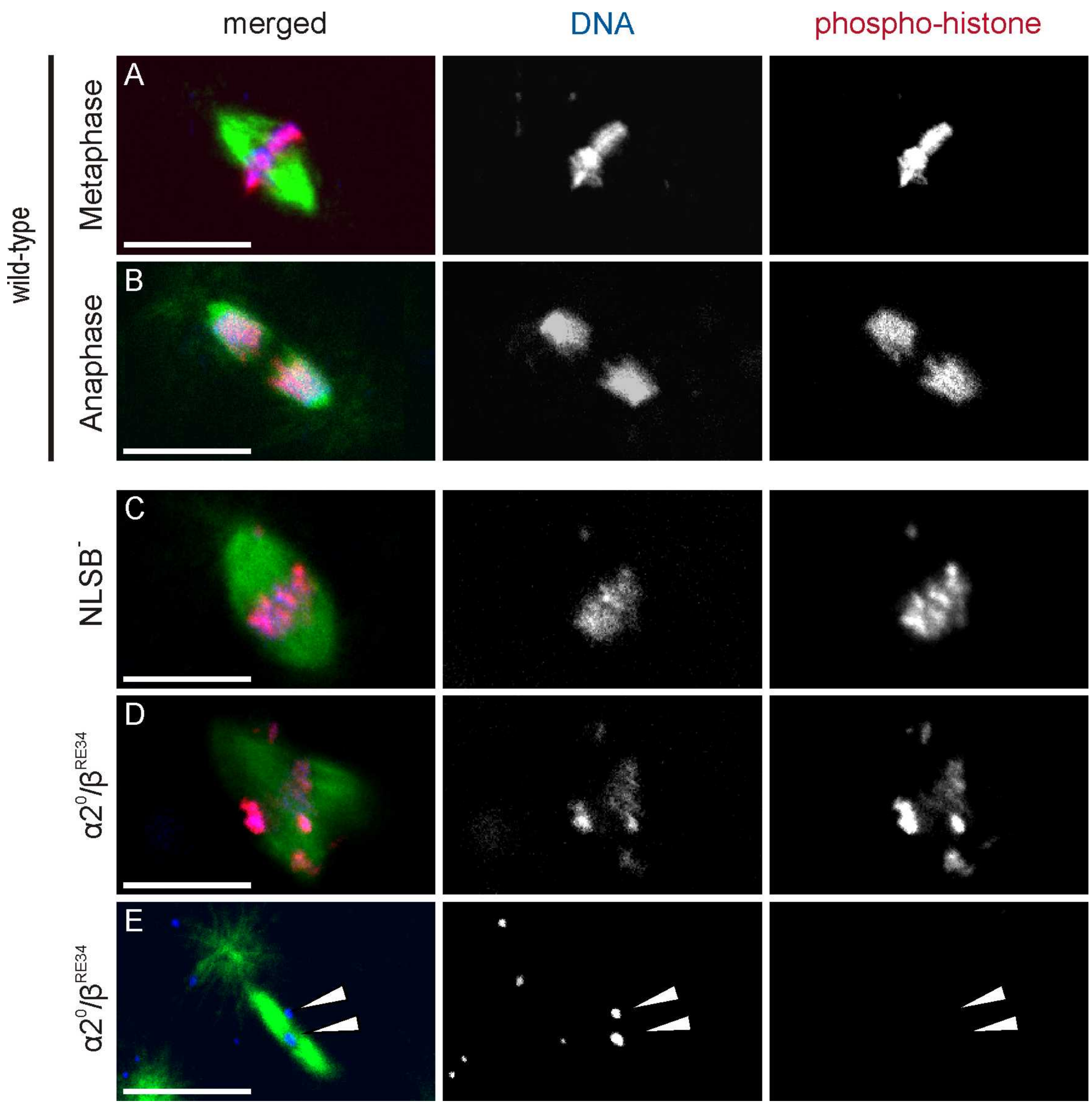

Figure S4 Chromosome abnormalities in phospho-histone-stained embryos from *imp-α2*^{D14}*/imp-β*^{KetRE34} (*α2*^{0}*/β*^{RE34}) and *imp-α2*^{D14}*/imp-β*^{c02743}*; NLSB*$^{-}$*/+* (NLSB^{-}) mutant females. (A, B) Metaphase and anaphase spindles (resp.) in wild-type embryo. (C-E) Spindles in embryos derived from mutant females. (C) Phospho-histone positive, condensed chromosomes aligned at the metaphase-plate of a fatty spindle. (D) Phospho-histone positive chromosomes irregularly scattered on a multipolar spindle. (E) Phospho-histone negative, non-condensed chromatin fragments (arrowheads) on a narrow spindle. α-tubulin (green), phospho-histone (condensed mitotic DNA, red) and DNA (blue). Scale bar: 10µm.
