# Supplementary material for: Specific Cooperation Between Imp-α2 and Imp-β/Ketel in Spindle Assembly During Drosophila Early Nuclear Divisions
Source: G3 (Bethesda). 2012 Jan 1;2(1):1–14. doi: 10.1534/g3.111.001073 (PMC3276186; doi:10.1534/g3.111.001073)
Supplement: Supporting Information [file supp_2.1.1_FigureS5.pdf]

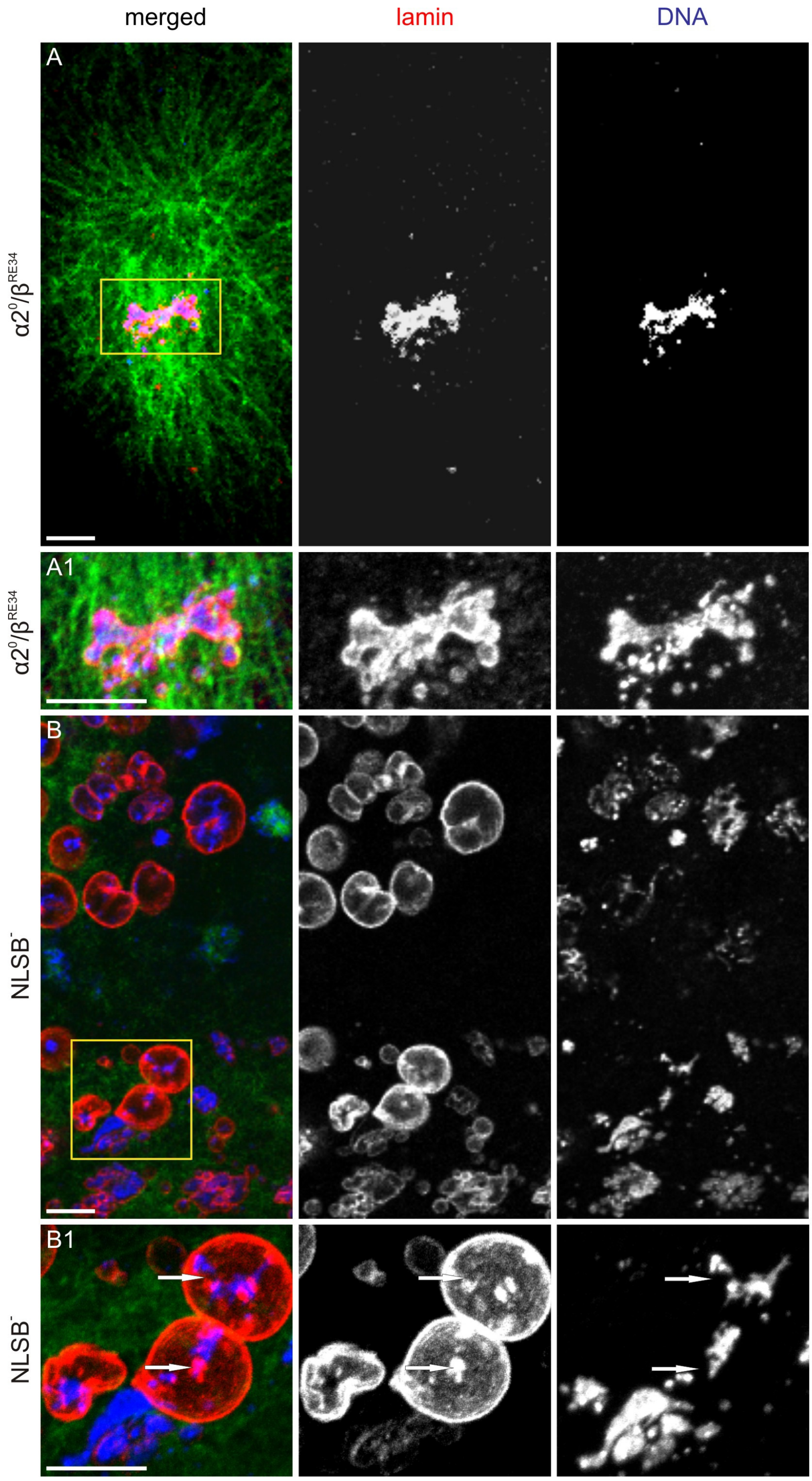

Figure S5 Defects of nuclear envelope assembly in embryos from *imp-α2*^{D14}*/imp-β*^{KetRE34} (*α2*^{0}*/β*^{RE34}) and *imp-α2*^{D14}*/imp-β*^{c02743}*; NLSB*$^{-}$*/+* (NLSB^{-}) females. (A, A1) Chromatin fragments surrounded by lamin on the background of an aster-like accumulation of microtubules. (B, B1) Abnormally large lamin spheres enclosing chromatin fragments and lamin aggregates (arrows). α-tubulin (green), lamin Dm0 (red) and DNA (blue). Scale bar: 10µm.
