# Supplementary material for: Specific Cooperation Between Imp-α2 and Imp-β/Ketel in Spindle Assembly During Drosophila Early Nuclear Divisions
Source: G3 (Bethesda). 2012 Jan 1;2(1):1–14. doi: 10.1534/g3.111.001073 (PMC3276186; doi:10.1534/g3.111.001073)
Supplement: Supporting Information [file supp_2.1.1_TableS1.pdf]

Table S1 RNAi silencing of *imp-α2* in heterozygous *imp-β*^{KetRE34} females strongly reduces egg viability.

Female genotype	Egg viability (%)	SD	n
imp-α2^{D14}*/+*	89	2.49	285
imp-β^{KetRE34}*/+*	61	9.50	234
P{imp-α2i}/ nos-Gal4^{VP16}	93	3.42	150
imp-α2^{D14}*/+; P{imp-α2i}/ nos-Gal4*^{VP16}	57	4,55	176
imp-β^{KetRE34}*/+; P{imp-α2i}/ nos-Gal4*^{VP16}	3	1.73	530

SD=standard deviation, n=number of embryos scored
