# Supplementary material for: Specific Cooperation Between Imp-α2 and Imp-β/Ketel in Spindle Assembly During Drosophila Early Nuclear Divisions
Source: G3 (Bethesda). 2012 Jan 1;2(1):1–14. doi: 10.1534/g3.111.001073 (PMC3276186; doi:10.1534/g3.111.001073)
Supplement: Supporting Information [file supp_2.1.1_TableS2.pdf]

Table S2 Effect of $D^{725}N$ substitution on docking energy of the IBB domain of Imp-α2 as a ligand on Imp-β as a receptor

Receptor	Ligand[a]	Lowest docking energy [kcal/mole][b]
Imp-β	Imp-α2 IBB domain	-75.63
Imp-β^{D725N}	Imp-α2 IBB domain	-75.53

Molecular structures of wild-type Drosophila Imp-β and the IBB (Importin Beta Binding) domain of wild-type Drosophila Imp-α2 are according to that of detected for human Imp-β complexed with the IBB domain of human Imp-α (PDB – ID: 1QGK). The structure of Drosophila Imp-β^{D725N} is generated by homology modelling using that of the wild type as template.

[a] Residues 17-53 of the IBB domain in helical conformation.
[b] Lowest values of 100 dockings.
