# Supplementary material for: Specific Cooperation Between Imp-α2 and Imp-β/Ketel in Spindle Assembly During Drosophila Early Nuclear Divisions
Source: G3 (Bethesda). 2012 Jan 1;2(1):1–14. doi: 10.1534/g3.111.001073 (PMC3276186; doi:10.1534/g3.111.001073)
Supplement: Supporting Information [file supp_2.1.1_TableS3.pdf]

Table S3 Effects of RNAi silencing of the three *imp-α* genes on embryo viability in combination with *imp-β*^{KetRE34}

Female genotype	Egg viability (%)	SD	n
imp-β^{KetRE34}*/+*	61	9.50	234
imp-β^{KetRE34}*/ P{imp-α1i}; nos-Gal4*^{VP16}*/+*	40	5.03	254
imp-β^{KetRE34}*/+; P{imp-α2i*^{34265}*}/ nos-Gal4*^{VP16}	0	0.00	610
imp-β^{KetRE34}*/+; P{imp-α2i*^{34266}*}/ nos-Gal4*^{VP16}	3	3.07	531
P{imp-α3i^{36104}*}/+; imp-β*^{KetRE34}*/+; nos-Gal4*^{VP16}*/+*	44	2.64	250
imp-β^{KetRE34}*/ P{imp-α3i*^{36103}*}; nos-Gal4*^{VP16}*/+*	43	2.82	123

SD=standard deviation, n=number of embryos scored
